# Naringin and temozolomide combination suppressed the growth of glioblastoma cells by promoting cell apoptosis: network pharmacology, *in-vitro* assays and metabolomics based study

**DOI:** 10.3389/fphar.2024.1431085

**Published:** 2024-07-30

**Authors:** Priya Bisht, Surendra Rajit Prasad, Khushboo Choudhary, Ruchi Pandey, Dande Aishwarya, Vulli Aravind, Peraman Ramalingam, Ravichandiran Velayutham, Nitesh Kumar

**Affiliations:** ^1^ Department of Pharmacology and Toxicology, National Institute of Pharmaceutical Education and Research (NIPER-Hajipur), Export Promotion Industrial Park (EPIP), Hajipur, Bihar, India; ^2^ Department of Biotechnology, National Institute of Pharmaceutical Education and Research (NIPER-Hajipur), Export Promotion Industrial Park (EPIP), Hajipur, Bihar, India; ^3^ Department of Pharmaceutical Analysis, National Institute of Pharmaceutical Education and Research (NIPER-Hajipur), Export Promotion Industrial Park (EPIP), Hajipur, Bihar, India

**Keywords:** glioblastoma, TMZ resistance, PARP-1, naringin, network pharmacology, chemosensitization, apoptosis, metabolomics

## Abstract

**Introduction:** Glioblastoma, which affects a large number of patients every year and has an average overall lifespan of around 14.6 months following diagnosis stands out as the most lethal primary invasive brain tumor. Currently, surgery, radiation, and chemotherapy with temozolomide (TMZ) are the three major clinical treatment approaches. However, the ability to treat patients effectively is usually limited by TMZ resistance. Naringin, a bioflavonoid with anti-cancer, antioxidant, metal-chelating, and lipid-lowering effects, has emerged as a promising therapeutic option.

**Methods:** To explore the targets and pathways of naringin and TMZ in glioblastoma network pharmacology, cell line-based ELISA, flow cytometry, immunocytochemistry, western blotting, and LC-HRMS based metabolomics study were used.

**Results:** The findings through the network pharmacology suggested that the key targets of naringin in the chemosensitization of glioblastoma would be Poly [ADP-ribose] polymerase 1 (PARP-1), O-6-Methylguanine-DNA Methyltransferase (MGMT), and caspases. The functional enrichment analysis revealed that these targets were significantly enriched in important pathways such as p53 signaling, apoptosis, and DNA sensing. Further, the results of the *in-vitro* study in U87-MG and T98-G glioblastoma cells demonstrated that TMZ and naringin together significantly reduced the percentage of viability and inhibited the DNA repair enzymes PARP-1 and MGMT, and PI3K/AKT which led to chemosensitization and, in turn, induced apoptosis, which was indicated by increased p53, caspase-3 expression and decreased Bcl2 expression. Additionally, a metabolomics study in T98-G glioblastoma cells using liquid chromatography high-resolution mass spectrometry (LC-HRMS) revealed downregulation of C8-Carnitine (−2.79), L-Hexanoylcarnitine (−4.46), DL-Carnitine (−2.46), Acetyl-L-carnitine (−3.12), Adenine (−1.3), Choline (−2.07), Propionylcarnitine (−1.69), Creatine (−1.33), Adenosine (−0.84), Spermine (−1.42), and upregulation of Palmitic Acid (+1.03) and Sphingosine (+0.89) in the naringin and TMZ treatment groups.

**Discussion:** In conclusion, it can be said that naringin in combination with TMZ chemosensitized TMZ antiglioma response and induced apoptosis in tumor cells.

## 1 Introduction

The prognosis for people diagnosed with glioblastoma, the most malignant primary central nervous system (CNS) tumor, is frequently dismal. Despite extensive therapeutic investigation, there are currently no curable treatments available for glioblastoma, therefore the overall survival of glioblastoma patients is relatively poor. The shortest life expectancy rate for invasive brain tumors is less than 6% at 5 years while the average lifespan is 15–23 months (Ostrom et al., 2016). When glioblastoma is diagnosed, it is treated with the maximum amount of surgical excision, radiation, and temozolomide (TMZ) (Bisht et al., 2022). Owing to the invading characteristic of glioblastoma, surgical removal of the tumor barely eradicates all tumor cells, necessitating post-operative care to avoid recurrence. TMZ was initially discovered in the 1970s. The FDA approved the DNA-alkylating drug TMZ in 2005 for the treatment of newly identified brain tumors. Five of the 10 patients who underwent adjuvant TMZ chemotherapy in the first clinical study using TMZ in 1993 demonstrated appreciable clinical and radiological improvement. The first study’s results encouraged other effective investigations of TMZ chemotherapy in patients with GBM (Shergalis et al., 2018). However, TMZ resistance often poses a barrier to successful treatment (Gupta et al., 2019; Singh et al., 2021).

TMZ is an imidazole derivative with a molecular weight of 194.15. This is a second-generation alkylating chemotherapeutic drug originally part of a rational drug discovery program in the 1980s. As TMZ is lipid soluble, it penetrates the blood-brain barrier (BBB) quickly and thus becomes accessible within the CNS. At acidic pH levels, it is stable. However, quickly hydrolyzes to its active form (5-(3-methyltriazen-1-yl) imidazole-4-carboxamide: MTIC) intermediate at alkaline and neutral pH (>7) levels. The active form, methyl diazonium ion, is produced *in situ* and adds the methyl group to the bases of DNA (Barciszewska et al., 2015). Adenine’s N3 site (m3A) and the N7 site of guanine (m7G) account for 9% and 70%, respectively, of adduct formation, and the O6 site of guanine (m6G), is the site of methylation that occurs most frequently. O6-methylguanine (MeG), despite being the least often occurring methylated DNA adduct, is a significant cytotoxic adduct caused due to TMZ chemotherapy. The principal mechanism through which TMZ resistance is induced involves the activation of several DNA repair mechanisms such as base-excision repair (BER) and O6-methylguanine-DNA methyltransferase (MGMT). Nevertheless, a multitude of other processes, such as aberrant signaling pathways, autophagy, extracellular vesicle formation, and microRNAs, also play a role in the development of resistance to TMZ. (Barciszewska et al., 2015; Bisht et al., 2022).

Flavonoids are naturally occurring compounds having pharmacological properties that have seen a sharp upsurge in research conducted in the discipline of medicine with a therapeutic emphasis (Mahmud et al., 2023). Because of its strong anti-cancer, antioxidant, metal-chelating, and lipid-lowering properties, naringin, a flavanone-7-O-glycoside, has been a hot candidate in the therapeutics arena (Raja Kumar et al., 2019; Salehi et al., 2019). Naringin is present in abundance naturally in grapefruits, and citrus, where it regulates the bitter taste of these fruits (Shilpa et al., 2023).

Drug development has significant time and financial expenditures. A novel technique for comprehensively evaluating and forecasting the mechanisms of an active ingredient or medication is network pharmacology. It evolved by replacing the antiquated “one target, one drug” investigation approach with the more recent “network target, multi-components” paradigm. Initially prospective targets for an active chemical are chosen from several databases, and then genetic data from the disease database is incorporated. Following that, to aid in mechanistic research or the creation of novel drugs, a functional network is created based on the gene enrichment analysis findings. Rational and economical drug development depends on this strategy since it significantly lowers the drug attrition rate. It is also appropriate for investigating current mechanisms to develop novel drugs (Shi et al., 2022). In a comparable vein, we have attempted to methodically assess the mechanism of naringin in glioblastoma through data analysis. In the current study, we initially extracted the target genes that correspond to naringin. Next, we identify genes from the disease database and carry out relevant analyses and enrichment. Thirdly, we integrate the aforementioned outcome into a network. Lastly, a naringin and TMZ-induced glioblastoma model was created to conduct further validation.

## 2 Material and methods

### 2.1 Network pharmacology

#### 2.1.1 Possible targets of naringin

The predicted targets of naringin were obtained using the database: Swiss Target Prediction (http://www.swisstargetprediction.ch/). Swiss Target Prediction is a database that predicts the targets of active compounds using combined 2D or 3D similarity measures with well-known ligands (Gfeller et al., 2014).

#### 2.1.2 Possible targets of glioblastoma

The following database was used to gather information on glioblastoma-associated target genes. DisGeNET (https://www.disgenet.org/search) is a multi-functional database that integrates genes, disease, and experimental studies (Yu et al., 2019). As a result, we used the keywords “Glioblastoma” in DisGeNET to screen for targets related to glioblastoma and compared them with the potential target genes of naringin.

#### 2.1.3 PPI network map of compound-disease common targets

The protein-protein interaction (PPI) network was constructed using the STRING (https://string-db.org/) (Date of accession: 25 June 2024) database, which included almost all functional interactions between the expressed proteins. The species is set to “*Homo sapiens*” and the target interaction was determined based on the analysis results (Huang et al., 2019). Further to reduce the network’s complexity, unconnected nodes were concealed and the lowest needed interaction score was set at 0.700. The STRING software intends to incorporate all identified as well as predicted PPI, including both functional associations and physical interactions. STRING accomplishes this by gathering and scoring evidence from a variety of sources, including databases of interaction experimentations or annotated complexes/pathways, systematic transfers of interaction evidence from one species to another, and automated content mining of scientific papers (Szklarczyk et al., 2021).

#### 2.1.4 Active compound-target network

To represent the complex interaction between active compound and possible targets, the Cytoscape (version 3.10.2) (https://cytoscape.org/) (Date of accession: 3 April 2024) tool was used to create a visual network (Qin et al., 2020). Active compound and targets are represented by nodes, while intermolecular interactions between active compound and targets are represented by edges. Cytoscape is an open-source software application for analyzing and visualizing biological networks (Nishida et al., 2014).

#### 2.1.5 Assessment of Gene Ontology (GO) and Kyoto Encyclopedia of Genes and Genomes (KEGG) pathway

DAVID Knowlegebase (v2023q4) (https://david.ncifcrf.gov/) (Date of accession: 6 April 2024) was used to analyze Gene Ontology (GO) enrichment and Kyoto Encyclopedia of Genes and Genomes (KEGG) pathway enrichment, which provides the biological function and potential mechanisms of detected targets. The bioinformatics online analysis platform (https://bioinformatics.com.cn/en) (Date of accession: 6 April 2024) was used to visualize the enrichment analysis results (Nishida et al., 2014).

### 2.2 *In-vitro* study

#### 2.2.1 Cell culture

In the present study, the U87-MG (passage no: 22) and T98-G (CRL-1690) cell lines were used. The cell line was kept in Minimum Essential Medium (MEM) (Cat # 42360099) and Eagle’s Minimum Essential Medium (EMEM) (Cat # 30-2003), with 10% Fetal Bovine Serum (FBS) (Cat # A5209402), and 0.5% Antibiotic-Antimycotic antibiotic (Cat # 15240062) under sterile conditions at the cell culture lab at NIPER, Hajipur. In a humid incubator, cells were kept at 37°C with 5% CO_2_ supplementation. T98-G cell line and EMEM media were procured from ATCC; Manassas, VA, United States. U87-MG cell line was procured from the National centre for cell science (NCCS), Pune, Maharashtra, India. MEM media, FBS, and Antibiotic-Antimycotic antibiotic were procured from Gibco®; Thermo Fisher Scientific, Grand Island, NY, United States. Naringin (Cat #N0073) and Temozolomide (Cat #T2744) were procured from Tokyo Chemical Industry Co. Ltd. (TCI) chemicals, Saitama, Japan.

#### 2.2.2 Cytotoxicity assay

The MTT test has been used to assess the cytotoxicity of the drugs on U87-MG (human glioblastoma cell line) and T98-G cells (TMZ-resistant human glioblastoma cell line). Individual 96-well plates with a cell suspension of 1 × 10^5^ cells/mL in MEM for U87-MG cells, and in EMEM for T98-G cells were seeded, and the cells were then kept for 24 h. The following drugs were added to the wells after a phosphate buffer saline (PBS) (cat # ML023-500ML, Himedia, Mumbai, Maharashtra, India) wash, and they were then incubated for 48 h. Control (no drug), TMZ (15.62, 31.25, 62.5, 125, 250, 500, 1,000 μM) and naringin (15.62, 31.25, 62.5, 125, 250, 500, 1,000 μM) in both the plates (U87-MG and T98-G cells) and naringin + TMZ (15.62 + 15.62, 31.25 + 31.25, 62.5 + 62.5, 125 + 125, 250 + 250, 500 + 500, 1,000 + 1,000 μM) only in U87-MG cells plate (Rao et al., 2021). After that, 10 μL of 3-(4,5-dimethylthiazol-2-yl)-2,5-diphenyltetrazolium bromide (MTT) (cat #M6494, Invitrogen, Maryland, United States) at 0.5 mg/mL concentration was added into the wells and were kept at 37°C for 4 h. Following that, 100 μL of dimethyl sulfoxide (DMSO) (cat # MB058S, Hi media solutions, Mumbai, Maharashtra, India) was added, and the plate was shaken for 30 min. Using a multimode plate reader (Spectra Max id5), the absorbance was recorded at 570 nm (Sangaran et al., 2021) and the half-maximal inhibitory concentration (IC50) was obtained by nonlinear regression via GraphPad Prism application (version 8.0.1). After calculating the IC50 values of naringin and TMZ in both cell lines, MTT was performed in T98-G cells using the same procedure mentioned above. The following drug concentration was used. Control (no drug), naringin (243 μM; IC50 calculated in T98-G cells), TMZ (212.5 μM; IC50 calculated in U87-MG cells), and naringin + TMZ (243 + 212.5 μM).

#### 2.2.3 Estimation of the DNA repair enzyme (PARP-1, MGMT) concentration

U87-MG and T98-G (1×10^6^ cells/mL) cells were cultured for 24 h in a plate with six wells and then treated with naringin, TMZ, and naringin + TMZ for 24 h. After that, the supernatant was collected and centrifuged for 20 min to remove insoluble impurities and cell debris at 1,000 × g at 2°C–8°C. The clear supernatant was collected and the assay was carried out immediately as per the protocol described in the human Poly [ADP-ribose] polymerase 1 (PARP-1) (cat # ab285289) and O-6-Methylguanine-DNA Methyltransferase (MGMT) ELISA assay kit (cat # ab284030) (Abcam, Cambridge, MA). Using a multimode plate reader (Spectra Max id5), the absorbance was recorded at 450 nm.

#### 2.2.4 Wound scratch assay

CytoSelect 24-well wound healing assay kit (cat #CBA-120) (Cell Biolabs, Inc., United States.) was utilized as per the specifications provided by the manufacturer (Pecora et al., 2022) to study the effect of naringin, TMZ, and naringin + TMZ on the migration of T98-G cells. A suspension of T98-G cells including 1 × 10^6^ cells/mL in media with 10% (v/v) FBS was made. After that, with the insert in place, 500 μL of cell suspension was incorporated into each well, and the cells were incubated at 37°C in a 5% CO_2_ till a single layer of cells appeared. The insert was carefully withdrawn to create a wound field with a 0.9 mm gap between the cells. The media was then carefully drained from each well and thrown away. Then the wells were rinsed with new media to eliminate debris and dead cells. Upon washing, cells were treated with naringin, TMZ, and naringin + TMZ and subsequently kept at 37°C in a 5% CO_2_ environment. T98-G cell migration was tracked using an Axio Vert. A1 (Carl Zeiss, Germany) at 0, and 24 h, and pictures were captured with Zen blue software for image collection.

#### 2.2.5 Apoptosis

The AV apoptosis detection kit (cat # APOAF-20TST) (Sigma Aldrich, Burlington, Massachusetts, United States) was used to analyze cell binding to annexin V-FITC (AV)/propidium iodide (PI) (Saha et al., 2022). U87-MG and T98-G cells (1×10^6^ cells/mL) were cultured for 24 h in a plate with six wells and then treated with naringin, TMZ, and naringin + TMZ for 24 h. Cells were extracted, washed, and resuspended in 1 mL PBS before being mixed with 5 μL PI, 20 μL binding buffer, and 5 μL AV in that sequence. Following a 15-min (min) incubation period, cells were examined using a BD FACS Aria Fusion.

#### 2.2.6 Cell cycle

U87-MG and T98-G (1×10^6^ cells/mL) cells were cultured for 24 h in a plate with six wells and then treated with naringin, TMZ, and naringin + TMZ for 24 h. After trypsinization, cells were removed, washed with PBS, and fixed with ethanol at 4°C for 2 h. PBS was used to wash the fixed cells before staining them in the dark with 500 μL of FxCycle™ PI/RNase Staining Solution (cat #F10797) (Invitrogen, Maryland, United States) (Rojas-Barón et al., 2024). The cell cycle study was assessed using a BD FACS Aria Fusion.

#### 2.2.7 Protein expression using FACS

T98-G (1×10^6^ cells/mL) cell was cultured for 24 h in a plate with six wells and then treated with naringin, TMZ, and naringin + TMZ for 24 h. Cells were removed after trypsinization, washed in PBS, permeabilized for 1 h at 4°C with permeabilization solution (BD Bioscience, San Jose, CA), washed two times with wash buffer (BD Bioscience), and then stained for 1 h at 4°C with PARP-1 (1:100) (cat # BS-2138R), and MGMT (1:100) (cat # MA5-13506) (Invitrogen, Maryland, United States). Cells were then stained with the corresponding Alexa fluor 488 labeled anti-rabbit (cat # A-11029) and anti-mouse (cat # A32731) secondary antibodies (1:100) (Invitrogen, Maryland, United States) after being washed with wash buffer two times. Following that, the cells were washed two times using a wash buffer, and 300 μL wash buffer was added. The cells were then examined using BD FACS Aria Fusion (Jin and Lee, 2023).

#### 2.2.8 Western blotting

T98-G cells (1×10^6^ cells/mL) were seeded in a plate with six wells and then treated with naringin, TMZ, and naringin + TMZ for 24 h. Cells were removed after trypsinization and washed with PBS. 200 μL RIPA buffer (cat # TCL131, Himedia, Mumbai, Maharashtra, India) and protease inhibitor (cat #C756V54, Thomas Scientific, Swedesboro, NJ, United States) were added to cell pellets and cells were allowed to stand for 30 min. After that, cells were lysed using a probe sonicator. Further, the lysates were centrifuged at 10,000 g for 15 min at 4°C. A BCA protein assay kit (cat # 71285-3, MERCK, Burlington, Massachusetts, United States) was used to measure the concentration of protein. Laemmli buffer was added to 50 μg/sample, which was then heated for 5–10 min before being loaded onto (10%) SDS polyacrylamide gel (SDS-PAGE) and electrophoretically separated. The proteins were transferred to the polyvinylidene difluoride (PVDF) membrane (cat # 3010040001, Roche, Penzberg, Germany). After the transfer was completed, the membrane was blocked with bovine serum albumin (3% BSA in TBST) (cat # TC548 Himedia, Mumbai, Maharashtra, India) for an hour at room temperature (RT), and the proper dilutions of GAPDH (1:1,000, anti-mouse) (cat # MA5-37687), PARP-1 (1:1,000, anti-mouse) (cat # BS-2138R), p53 (1:1,000, anti-mouse) (cat # PA5-119490), Bcl-2 (1:50) (PA5-32327094), Phospho-PI3K (1:2000, anti-rabbit) (cat # PA5-104853) primary antibodies were then probed overnight at 4°C. The membranes were TBST-washed 5 times for 3 min before being kept for an hour with corresponding horseradish peroxidase (HRP) labeled secondary anti-rabbit (2:10,000) (cat # 31460) and antimouse (2:10,000) (cat # 31430) antibodies. The membranes were observed using ECL Western blot Chemiluminescence Reagent (cat # 32106) (Thermo Fisher Scientific, Grand Island, New York, United States) and the pictures were taken using a ChemiDoc system (Bio-Rad). Band intensities were first normalized to GAPDH as a loading control, and ImageJ 1.8.0_172 was then used to further normalize the data to control. All the primary and secondary antibodies were procured from Invitrogen, Maryland, United States.

#### 2.2.9 Immunocytochemistry

In a 6-well plate, U87-MG and T98-G 1×10^6^ cells/mL were cultured per well on coverslips and then treated with naringin, TMZ, and naringin + TMZ for 24 h. Following 24 h, the cells were washed thrice in PBS. Further, cells were permeabilized using methanol (cat # 79345) (Sisco Research Laboratories Pvt. Ltd. Maharashtra, India) for 20 min and blocked at RT for 1 h in PBS including 0.1% triton X-100 (cat # RM845-100 ML) (Himedia, Mumbai, Maharashtra, India), 0.1% tween 20 (cat # MB067-100 ML) (Himedia, Mumbai, Maharashtra, India) and 5% BSA. Antibodies of p53 (cat # PA5-119490), caspase-3 (cat # 43-7800), and bcl2 (cat # MA5-11757) were diluted 1:200, 1:1,000, and 1:100 accordingly, in PBS including 0.1% tween 20% and 1% BSA, and kept overnight at 4°C. After that, cells were washed thrice for 5 min in PBS and before being kept for 1 h with anti-mouse secondary antibody labeled with Alexa Fluor 488 (cat # A32731, diluted 1:500 in triton X-100, 0.1% tween 20% and 1% BSA in PBS). The cells were then washed thrice in PBS for 5 min before being stained with 1 μg/mL 4’,6-diamidino-2-phenylindole (DAPI) (cat # MBD0020) (MERCK, Burlington, Massachusetts, United States). Axio Vert. A1 Carl Zeiss fluorescent microscope with adequate filters and Zen blue application (Applied Imaging Corporation) was utilized for capturing pictures (Jin and Lee, 2023).

#### 2.2.10 Metabolomic profiling

##### 2.2.10.1 Sample preparation

Briefly, T98-G cells (1 × 10^6^ cells per well) were cultured and treated by naringin, TMZ, and naringin + TMZ for 24 h. To isolate metabolites, cells were collected with the use of trypsinization and washed with distilled PBS (twice). Cells were lysed as per the steps mentioned previously (under section 2.2.8). The supernatant acquired through this procedure was used in the extraction of metabolites. For every unit of the sample, three times the volume of chilled LC-HRMS grade acetonitrile (ACN) (cat # 9853-02, JT Baker, Matsonford road Radnor, PA) was added. Furthermore, the samples underwent incubation at −20°C for 60 min, followed by centrifugation at 15,000 *g* for 10 min (4°C). This process resulted in the collection of the supernatant, which was subsequently dried using a speedVac. Before LC-HRMS analysis, the dried samples were reconstituted in 300 μL of 0.1% formic acid (cat # A117-50, Thermo Fisher Scientific, Grand Island, NY) (Sheikh et al., 2011; Pan et al., 2021).

##### 2.2.10.2 Mass spectrometric analysis

The metabolite samples were analyzed using UHPLC (ThermoFisher Scientific Dionex Ultimate 3000 UHPLC + Focused), coupled with mass spectrometry (ThermoFisher Scientific Quadrupole(Q) -Orbitrap Exploris 240). Metabolite separation was accomplished using a Thermo Scientific Hypersil GOLD C18 column (100 × 2.1 mm, 1.9 µ) maintained at 40°C. The mobile phase consisted of solvent A (0.1% formic acid) and solvent B (100% acetonitrile, 0.1% formic acid). A gradient elution method was applied, with B% values set at 5% for 0.5 min, 95% from 0.6 to 22.0 min, and then returned to the initial condition in the last 8 min. The flow rate was set at 0.3 mL/min, and the injection volume was 20 μL. Throughout the analysis, all samples were kept at 4°C (autosampler temperature). Analysis was conducted in positive and negative ionization modes with a voltage of 4,000 V and 3,000 V, respectively. Metabolite MS/MS detection utilized Heated-electrospray ionization (H-ESI), with the ion transfer tube temperature set to 300°C and the vaporizer temperature set to 320°C. Sheath gas, auxiliary gas, and sweep gas were maintained at 40, 10, and 1, respectively. High-energy collision dissociation (HCD) collision energy was set at 10, 50, and 150% to obtain MS/MS spectra for identified metabolites. The mass range for full scan MS and MS/MS analysis was set from m/z 70 to 2,000. The S lens was maintained at 80%. Resolutions of 120,000 and 15,000 were maintained in full scan and ddMS two scans, respectively. The acquired data underwent processing using Compound Discoverer TM software, version 3.3, for metabolite identification and analysis. MetaboAnalyst 6.0 was employed for further data analysis and stratification.

##### 2.2.10.3 Data processing and analysis

RAW spectra of metabolite samples obtained after mass spec analysis were identified and analyzed using Compound Discoverer TM 3.3 (CD). We applied an established workflow designed for untargeted metabolomics, specifically the “Untargeted Metabolomics with statistics detect unknowns with ID using Online Database and mzLogic” workflow. The annotation of compounds at the MS/MS level with a mass tolerance of 5 ppm was conducted using McCloud. ChemSpider, which integrates BioCyc, chEMBl, and KEGG database, was utilized for annotating features based on exact mass with a mass tolerance of 5 ppm. Additionally, the CD internal database, housing 4,400 endogenous metabolites, was employed for annotation. The data matrices generated were subsequently exported to MS Office Excel 2010 (in.csv format) and employed for multivariate data analysis using MetaboAnalyst 6.0, a freely accessible web-based platform for metabolomics data processing (Xiong et al., 2020). Further, to elucidate the variations between the aforementioned groups, a supervised partial-least-squares-discriminate-analysis (PLS-DA) was employed as a means to ascertain the metabolites that exhibited significant impact in terms of the group. Model quality has been quantified using R2, representing model validity against overfitting, and Q2, which serves as an indicator of predictive ability. The identification of potential metabolite markers was achieved through the utilization of loading plots, specifically for PLSDA, and the application of the variable importance on projections (VIP) methodology. Metabolites statistical significance was assessed using a *t*-test (*p* < 0.05).

### 2.3 Statistical analysis

The D'Agostino-Pearson omnibus *post hoc* test was used to analyze the results’ normality of distribution. One-way analysis of variance (ANOVO) and Tukey’s test were applied for every possible comparison between the study groups, and the application GraphPad Prism (version 8.0.1) was used. The data were represented as mean ± SD and statistical significance for variations between group results was set at *p* < 0.05.

## 3 Results

### 3.1 Shared targets between naringin and glioblastoma

A total of 100 naringin targets were found through the Swiss Target Prediction and The Binding Database. By screening DisGeNET, 2,838 glioblastoma-related targets were obtained. By comparing the targets of naringin and glioblastoma, we discovered that glioblastoma shares 51 targets with naringin. Table 1 shows the shared targets between naringin and glioblastoma with their corresponding Uniprot IDs.

**TABLE 1:** Shared targets between naringin and glioblastoma with their corresponding Uniprot IDs.

Uniprot IDs	Targets
P11511	Cytochrome P450 19A1 (CYP19A1)
P31639	Sodium/glucose cotransporter 2 (SLC5A2)
P23219	Cyclooxygenase-1 (PTGS1)
P14679	Tyrosinase (TYR)
P03956	Matrix metalloproteinase 1 (MMP1)
P09237	Matrix metalloproteinase 7 (MMP7)
P45452	Matrix metalloproteinase 13 (MMP13)
P39900	Matrix metalloproteinase 12 (MMP12)
P27338	Monoamine oxidase B (MAOB)
Q9UNQ0	ATP-binding cassette sub-family G member 2 (ABCG2)
O43570	Carbonic anhydrase XII (CA12)
P33527	Multidrug resistance-associated protein 1 (ABCC1)
P16152	Carbonyl reductase [NADPH] 1 (CBR1)
P09874	Poly [ADP-ribose] polymerase-1 (PARP1)
P17706	T-cell protein-tyrosine phosphatase (PTPN2)
P09382	Galectin-1 (LGALS1)
P00918	Carbonic anhydrase II (CA2)
P08183	P-glycoprotein 1 (by homology) (ABCB1)
P03372	Estrogen receptor alpha (ESR1)
Q92731	Estrogen receptor beta (ESR2)
O14746	Telomerase reverse transcriptase (TERT)
P15121	Aldose reductase (AKR1B1)
Q9NUW8	Tyrosyl-DNA phosphodiesterase 1 (TDP1)
P25101	Endothelin receptor ET-A (by homology) (EDNRA)
P42574	Caspase-3 (CASP3)
Q14416	Metabotropic glutamate receptor 2 (by homology) (GRM2)
Q04609	Glutamate carboxypeptidase II (FOLH1)
P08254	Matrix metalloproteinase 3 (MMP3)
P78536	ADAM17 (ADAM17)
P49789	Bis(5′-adenosyl)-triphosphatase (FHIT)
P00519	Tyrosine-protein kinase ABL (ABL1)
P29317	Ephrin type-A receptor 2 (EPHA2)
Q06187	Tyrosine-protein kinase (BTK)
P24941	Cyclin-dependent kinase 2 (CDK2)
P06493	Cyclin-dependent kinase 1 (CDK1)
P01112	Transforming protein p21/H-Ras-1 (HRAS)
P56817	Beta-secretase 1 (BACE1)
Q14790	Caspase-8 (CASP8)
P29466	Caspase-1 (CASP1)
P18031	Protein-tyrosine phosphatase 1B (PTPN1)
P17936	Insulin-like growth factor binding protein 3 (IGFBP3)
P12821	Angiotensin-converting e494 vnzyme (ACE)
P55210	Caspase-7 (CASP7)
P42575	Caspase-2 (CASP2)
P05121	Plasminogen activator inhibitor-1 (SERPINE1)
P09038	Basic fibroblast growth factor (FGF2)
P02768	Serum albumin (ALB)
P08473	Neprilysin (MME)
P42892	Endothelin-converting enzyme 1 (ECE1)
O00214	Galectin-8 (LGALS8)
P16455	6-O-methylguanine-DNA methyltransferase (MGMT)

### 3.2 PPI network

**FIGURE 1:**
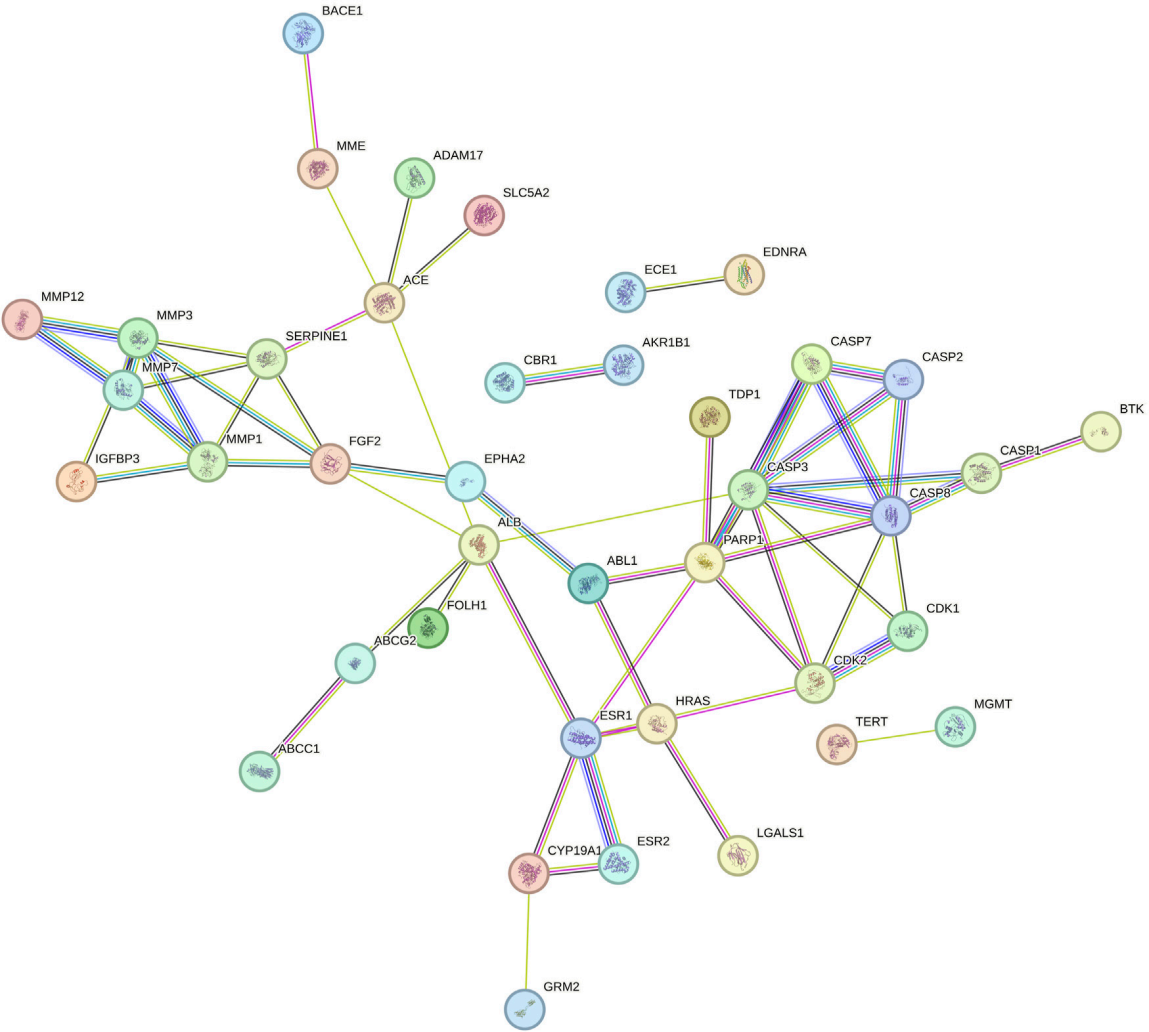
PPI network of shared targets between naringin and glioblastoma. Every bubble node reflects a protein, and the three-dimensional (3D) structure within the bubble nodes indicates whether the spatial structure of the protein is established or projected. The association between various proteins is shown by the lines dividing inner nodes, the width of which is determined by the degree of data validation. Abbreviation: PPI - Protein-protein interaction, 3D - three-dimensional.

The String database was used to perform protein-protein interaction network analysis on the constipation targets, and the PPI network was formed which is shown in Figure 1. A total of 51 nodes and 241 edges were obtained from the results, with an average node degree is 9.45.

### 3.3 Naringin-target network

**FIGURE 2:**
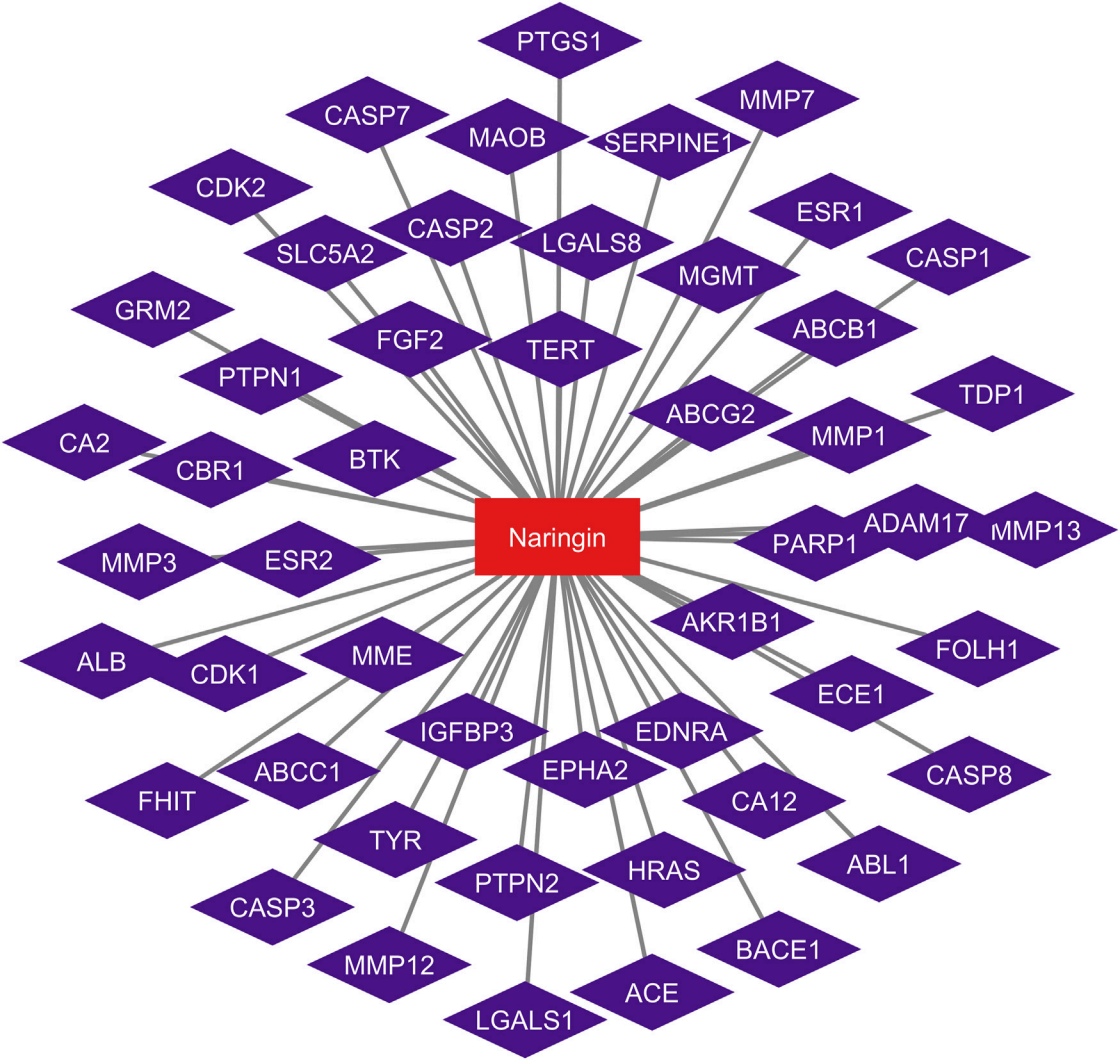
Naringin-target network of potential targets in glioblastoma. The red nodes represent the naringin, and the violet nodes represent the corresponding targets.

The network between naringin and glioblastoma is shown in Figure 2. Using Cytoscape, the network was built by mapping naringin and glioblastoma 51 potential targets. The network is embodied in 51 nodes and 50 edges, with colorized violet nodes corresponding to the targets and naringin in red.

### 3.4 Gene Ontology and KEGG enrichment analysis

**FIGURE 3:**
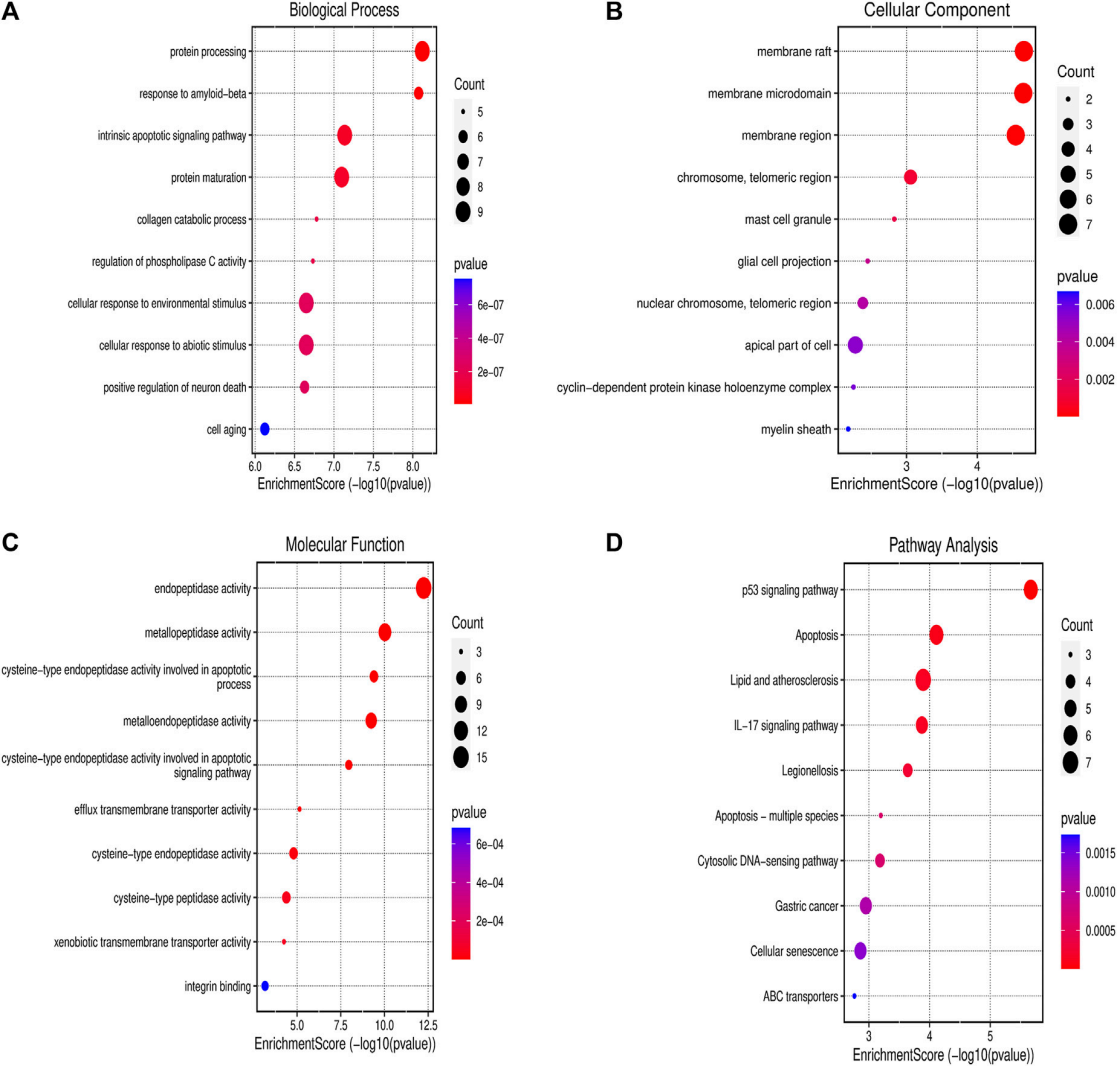
Gene Ontology and KEGG Enrichment Analysis **(A)** Biological process; **(B)** cellular component; **(C)** molecular function; and **(D)** KEGG. Abbreviation: KEGG - Kyoto Encyclopedia of Genes and Genomes.

The PPI network’s potential targets have been analyzed through the use of GO and KEGG enrichment analysis. The GO categorical results showed that these targets were involved in a number of biological processes, such as response to protein processing, intrinsic apoptotic signaling pathway, collagen catabolic process as well as a cellular response to the environment and abiotic stimulus protein processing (Figure 3A). The membrane raft, membrane microdomain, membrane region, chromosome, telomeric length, mast cell projection and glial cell projection were the primary components linked to their cellular function (Figure 3B). Furthermore, endopeptidase activity, metallopeptidase activity, cysteine-type endopeptidase activity involved in apoptotic process, metalloendopeptidase activity, cysteine-type endopeptidase activity involved in apoptotic signaling pathway were linked to the majority of targets in the PPI network (Figure 3C). Following KEGG enrichment analysis, the results showed that the signaling pathways may involve the p53 signaling pathway, apoptosis, lipid and atherosclerosis, IL-17 signaling pathway, and cytosolic DNA-sensing pathway (Figure 3D).

### 3.5 Cytotoxicity of naringin and TMZ in U87-MG and T98-G cells

**FIGURE 4:**
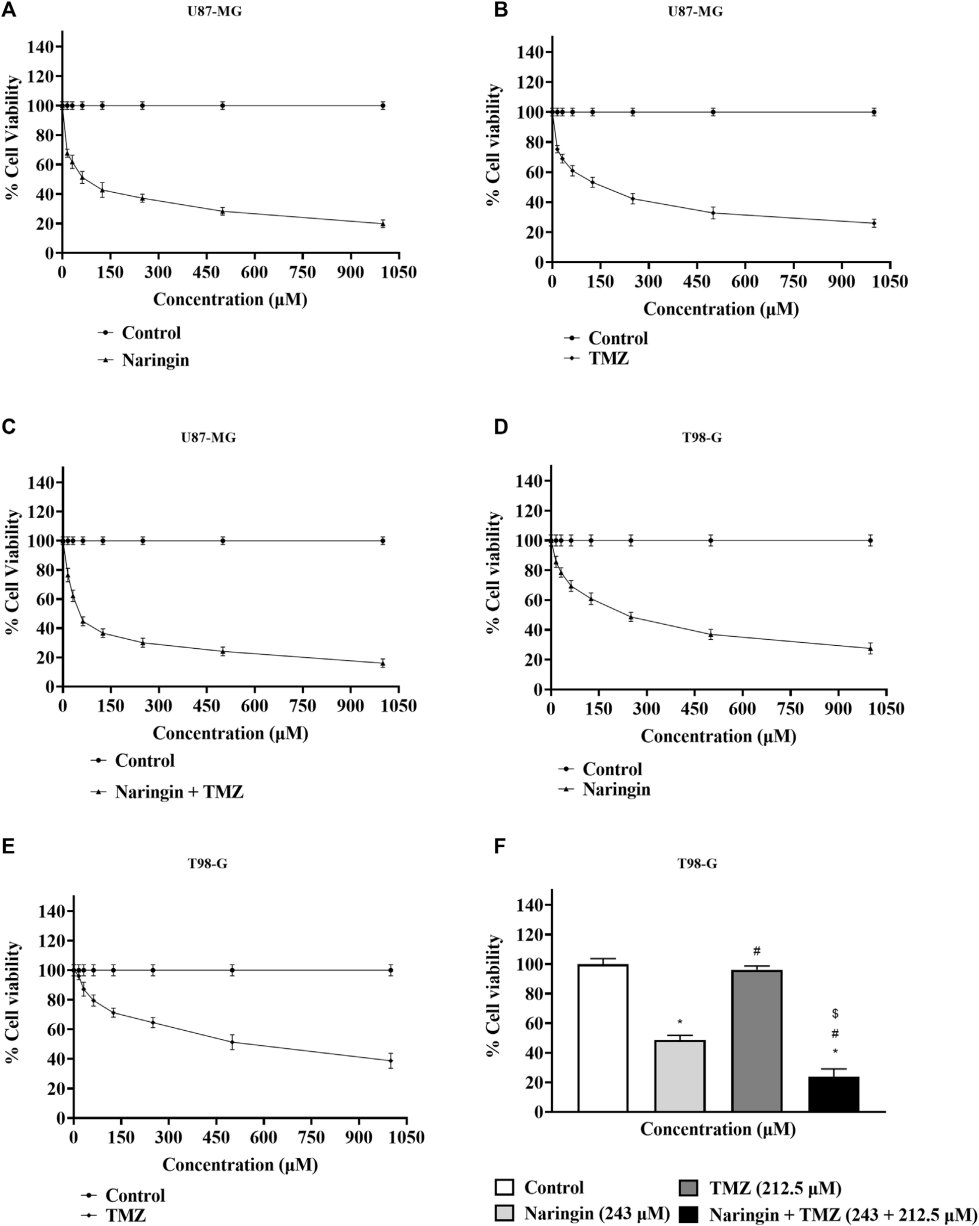
Percentage of cell viability by MTT test. **(A)** Percentage cell viability of naringin on the U87-MG cell line. **(B)** Percentage cell viability of TMZ on the U87-MG cell line. **(C)** Percentage cell viability of naringin + TMZ on the U87-MG cell line. **(D)** Percentage cell viability of naringin on the T98-G cell line. **(E)** Percentage cell viability of TMZ on the T98-G cell line. **(F)** Percentage cell viability of naringin, TMZ, and naringin + TMZ on the T98-G cell line. Results were presented as mean ± SD, n = 3. The D'Agostino-Pearson omnibus *post hoc* test was used to analyze the results’ normality of distribution. One-way ANOVA and Tukey’s multiple comparison tests were used for every possible comparison between the study groups. (*) *p* < 0.05, vs control; (^#^) *p* < 0.05 vs naringin; (^$^) *p* < 0.05 vs TMZ. Abbreviation: MTT - 3- [4,5-dimethylthiazol-2-yl]-2,5 diphenyl tetrazolium bromide, TMZ - Temozolomide.

The MTT test was performed to measure drug cytotoxicity and IC50 values in U87-MG and T98-G cells. The cytotoxicity assay was carried out in a dose-dependent manner. The calculated IC50 values for naringin on U87-MG cells were 121.3 μM (Figure 4A) and 243.0 μM on T98-G cells (Figure 4D). The calculated IC50 values for TMZ on U87-MG cells were 212.5 μM (Figure 4B) and 450.2 μM on T98-G cells (Figure 4E). The calculated IC50 values for naringin + TMZ on U87-MG cells were 38.14 μM (Figure 4C). MTT performed on T98-G cells at the following concentration: control (no drug), naringin (243 μM), TMZ (212.5 μM), and naringin + TMZ (243 + 212.5 μM), with no significant difference found between the control and TMZ groups, showing the chemoresistance of TMZ in TMZ resistance cells (T98-G). However, significant (*p* < 0.05) differences were found in the naringin and naringin + TMZ groups when compared to the control group. When naringin and TMZ were given in combination, there was a significant decrease in percentage viability, showing the chemosensitization of TMZ in TMZ resistance cells (T98-G) (Figure 4F).

### 3.6 Naringin and TMZ inhibited the DNA repair enzyme (PARP-1, MGMT) in U87-MG and T98-G cells

The PARP enzyme is a component of the BER complex, that consists of DNA ligase, XRCC1 as well as DNA polymerase ẞ all of which participate in the process of BER-mediated mechanism in response to SSBs in DNA. As a result, PARP represents a therapeutic potential in the modulation of the DNA repair system since it regulates SSBs. It has been demonstrated that targeted medicines that block PARP function dramatically increase the cytotoxicity of standard anti-cancer medications, including those intended for the treatment of GBM. PARP inhibitor drugs block the binding of PARP-MGMT or PARylation of MGMT, reducing MGMT function and preventing O^6^-MetG repair. As a result, the MGMT function is reduced. Moreover, we discovered PARP-1 and MGMT as possible naringin targets by network pharmacology. As a result, the effect of naringin and TMZ on PARP-1 and MGMT concentration must be determined.

**FIGURE 5:**
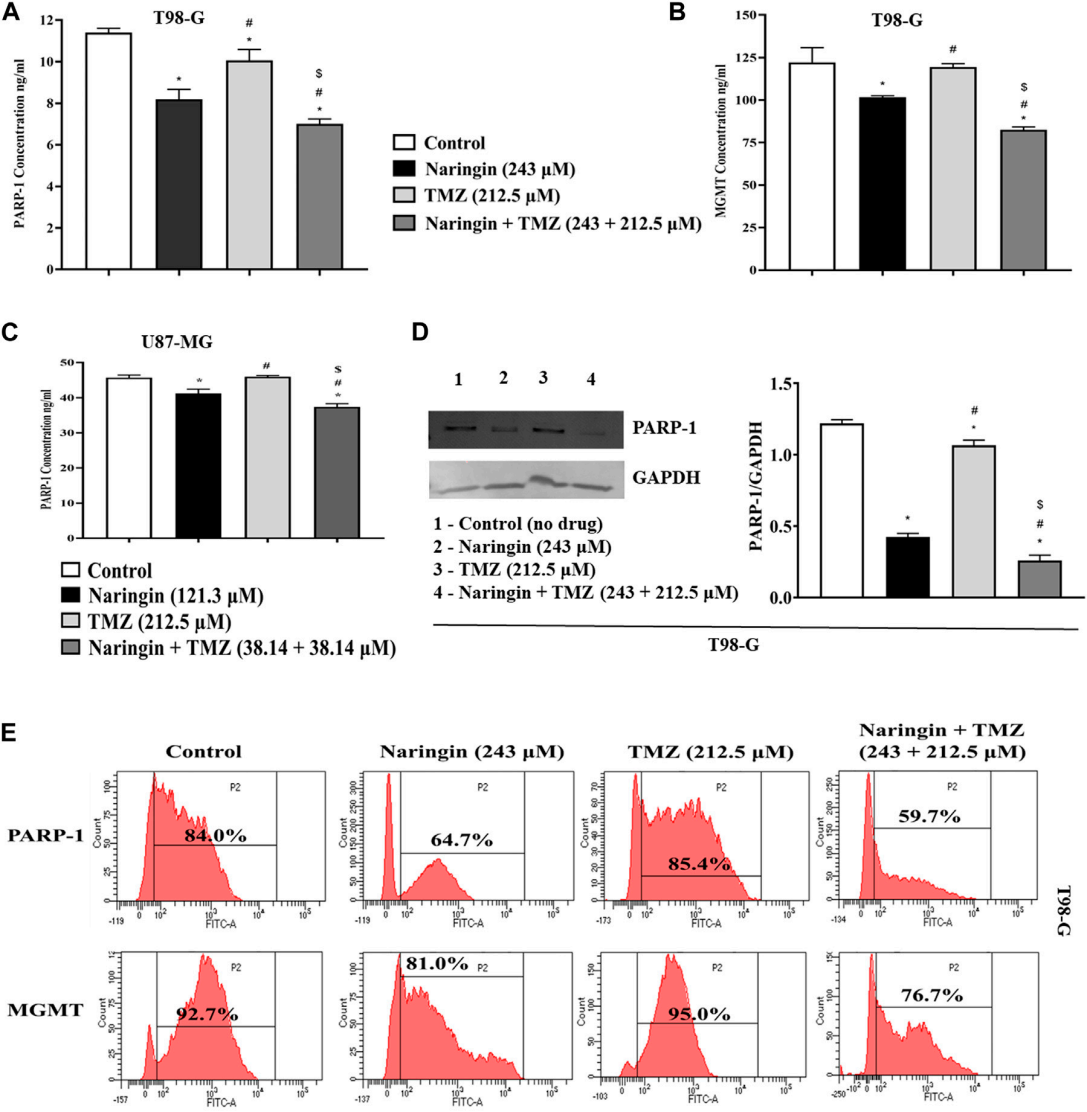
Assessment of PARP-1 and MGMT. **(A)** PARP-1 **(B)** MGMT (T98-G cells) **(C)** PARP-1 (U87-MG cells) concentration measurement by Elisa kit. **(D)** Expression of PARP-1 in T98-G cells by Western blotting. Lanes 1, 2, 3, and 4 are the control (no drug), naringin (243 μM), TMZ (212.5 μM), and naringin + TMZ (243 + 212.5 μM) groups, respectively, in the presentative Western blot picture of PARP-1 protein. The loading control used was GAPDH. For quantification of blots and images ImageJ software was used. **(E)** Expression of PARP-1 and MGMT by flow cytometry analysis. The percentage of protein expression is represented on the histogram. Results were presented as mean ± SD, n = 3. The D'Agostino-Pearson omnibus *post hoc* test was used to analyze the results’ normality of distribution. One-way ANOVA and Tukey’s multiple comparison tests were used for every possible comparison between the study groups. (*) *p* < 0.05, vs control; (^#^) *p* < 0.05 vs naringin; (^$^) *p* < 0.05 vs TMZ. Abbreviation: PARP-1 - Poly [ADP-ribose] polymerase 1, MGMT - O-6-Methylguanine-DNA Methyltransferase, TMZ - Temozolomide.

The concentration of PARP-1 is significantly (*p* < 0.05) reduced with pretreatment of TMZ at 212.5 μM, naringin at 243 μM and naringin + TMZ at 243 + 212.5 μM as compared to the control group. There is not much difference in the concentration of MGMT with pretreatment of TMZ at 212.5 μM while the concentration of PARP-1 and MGMT is significantly (*p* < 0.05) reduced with pretreatment of naringin at 243 μM and naringin + TMZ at 243 + 212.5 μM as compared to the control group (T98-G cells) (Figures 5A, B). The concentration of PARP-1 is significantly (*p* < 0.05) reduced with pretreatment of naringin at 121.3 μM and naringin + TMZ at 38.14 + 38.14 μM as compared to the control group. There is not much difference in the concentration of PARP-1 with pretreatment of TMZ at 212.5 μM (U87-MG cells) (Figure 5C).

### 3.7 Naringin and TMZ inhibited migration and invasion of T98-G cells

The *in-vitro* wound healing test was used in this work to examine how well naringin and TMZ slow down the regeneration process in T98-G cells. To do this, cells were treated with naringin (243 μM), TMZ (212.5 μM), and naringin + TMZ (243 + 212.5 μM), and their effects on migration and proliferation into the wound field were observed. Equations (1) and (2) were used for calculating the migration rate and the percentage of wound closure, accordingly:

$$\text{Percent of wound closure }\left(\%\right)=\frac{\text{Migrated}\;\text{cell}\;\text{surface}\;\text{area}}{\text{Total}\;\text{surface}\;\text{area}}\times 100$$

where migrated cell surface area is the length of cell migration (mm) × 2 × length and total surface area is equal to 0.9 mm × length;

$$\text{Migration rate}=\frac{\text{Length}\;\text{of}\;\text{cell}\;\text{migration}\;\left(\text{nm}\right)}{\text{Migration}\;\text{time}\;\left(h\right)}$$

**FIGURE 6:**
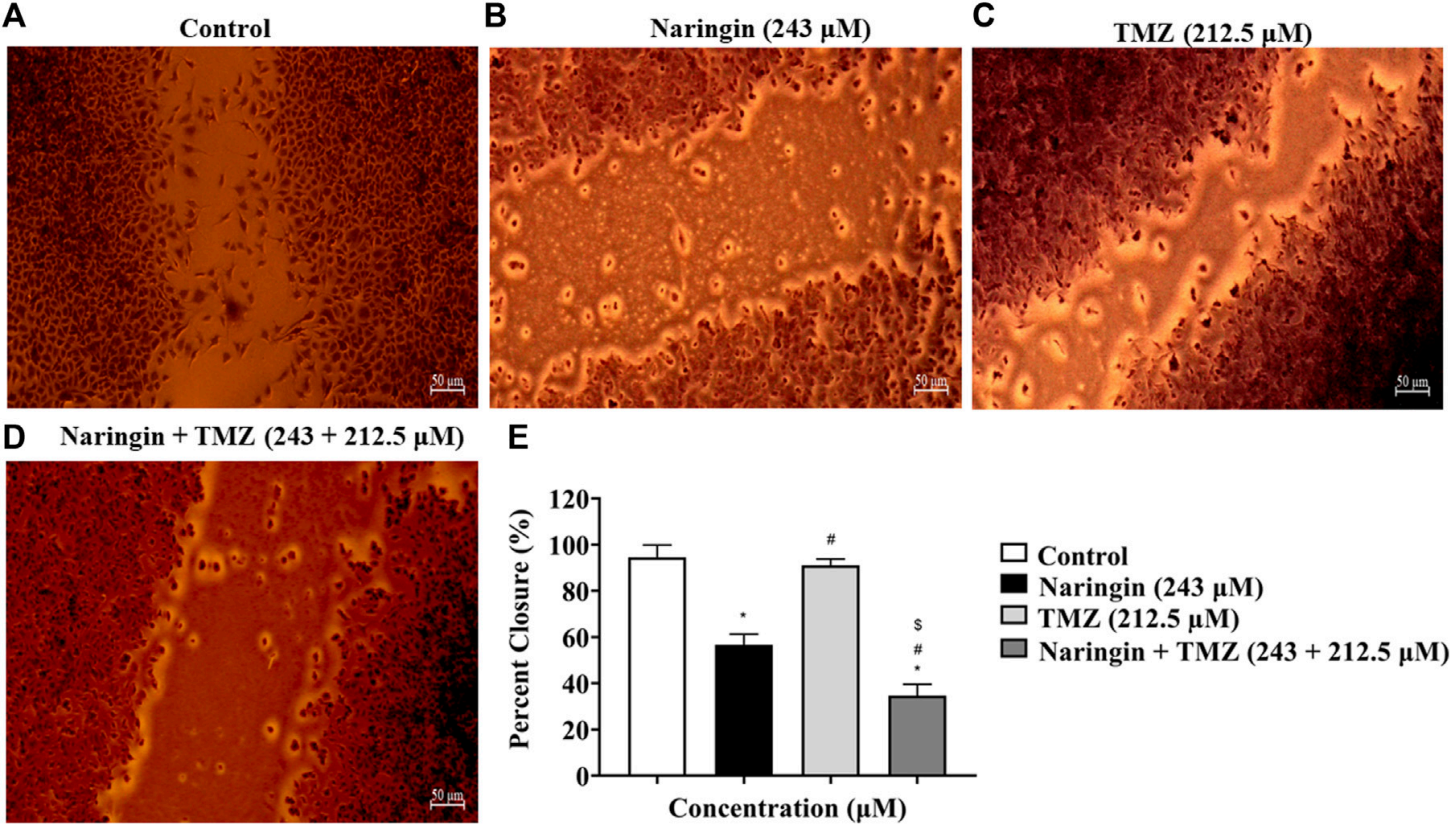
Percent wound closure by wound healing test. **(A)** Control (no drug), **(B)** naringin (243 μM), **(C)** TMZ (212.5 μM), and **(D)** naringin + TMZ (243 + 212.5 μM). **(E)** The percent wound closure represented on histograms calculated using ImageJ software. Results were presented as mean ± SD, n = 3. The D'Agostino-Pearson omnibus *post hoc* test was used to analyze the results’ normality of distribution. One-way ANOVA and Tukey’s multiple comparison tests were used for every possible comparison between the study groups. (*) *p* < 0.05, vs control; (^#^) *p* < 0.05 vs naringin; (^$^) *p* < 0.05 vs TMZ. Abbreviation: TMZ - Temozolomide.

At 24 h of treatment, the percent of wound closure was 94.59% in the control group, 56.62% in the naringin group, 91.11% in the TMZ group, and 34.66% in the naringin + TMZ group, and the migration rate was 35.43 ± 1.94 nm in the control group, 21.18 ± 1.73 in the naringin group, 34.15 ± 0.98 nm in the TMZ group, and 12.91 ± 1.84 mm in the naringin + TMZ group. There is not much difference in percent wound closure and migration rate between the control and TMZ groups, whereas a significant (*p* < 0.05) difference was seen in the naringin and naringin + TMZ combination groups as compared to the control group. The wound healing assay revealed that naringin and TMZ inhibited the migration activities of T98-G cells (Figure 6).

### 3.8 Naringin, TMZ and naringin + TMZ combination induce apoptosis in U87-MG and T98-G cells

**FIGURE 7:**
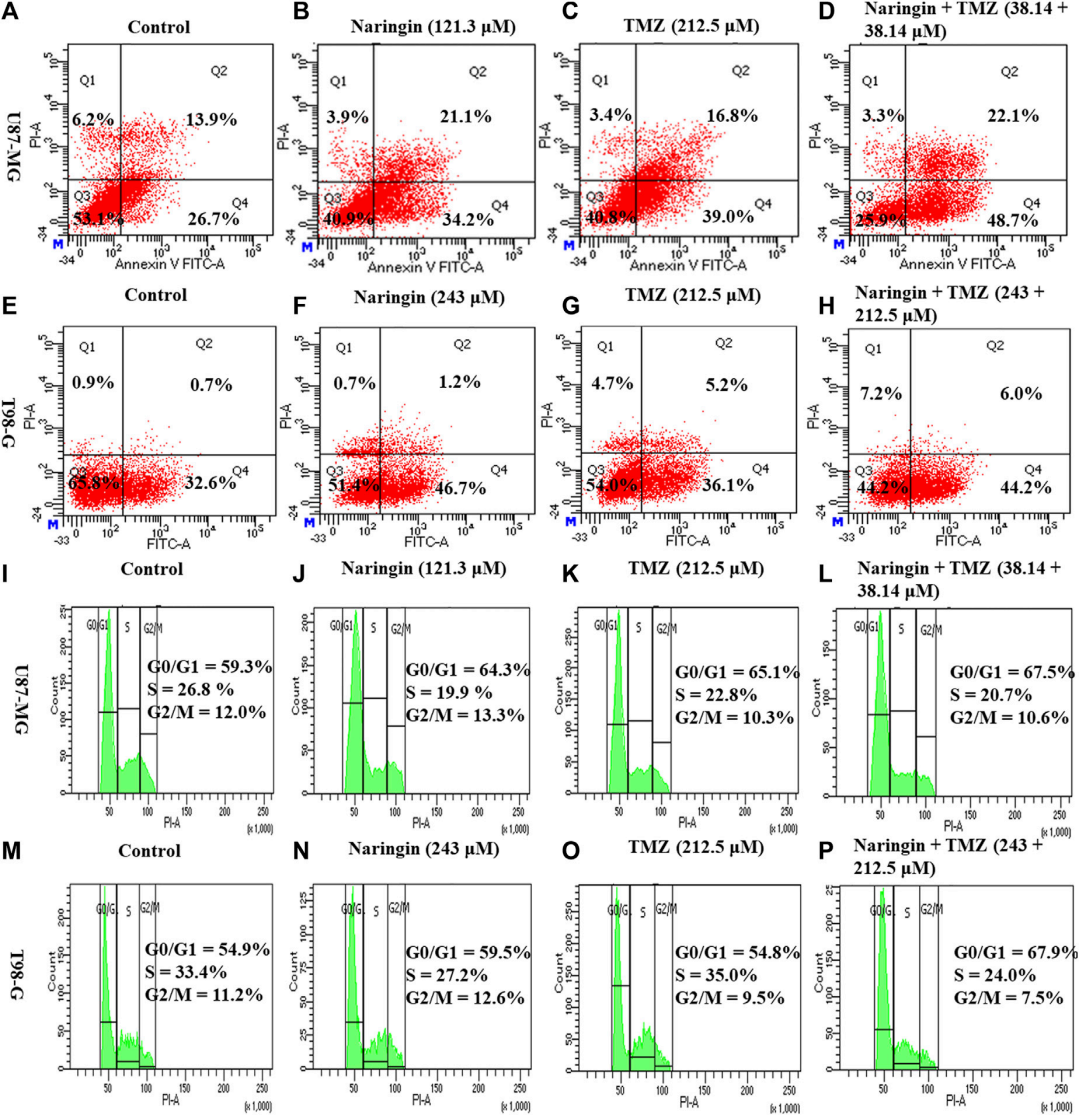
Apoptosis and cell cycle analysis of U87-MG and T98-G cells by Flow cytometry **(A–D)** Apoptosis in U87-MG cells; **(E–H)** apoptosis in T98-G cells; **(I–L)** cell cycle analysis in U87-MG cells; and **(M–P)** cell cycle analysis in T98-G cells. The percentage of apoptosis and cell cycle arrest is represented on histogram.

U87-MG cells were treated with naringin (121.3 μM), TMZ (212.5 μM), and naringin + TMZ (38.14 + 38.14 μM), while T98-G cells were treated with naringin (243 μM), TMZ (212.5 μM), and naringin + TMZ (243 + 212.5 μM). Annexin V was used to assess the apoptotic effect. The induction of apoptosis by naringin, TMZ, and the naringin + TMZ combination in both cell lines was observed using the AV/PI assay kit. Figures 7A–H shows FACS histograms with the proportion of each quadrant representing the apoptosis percentage of treated cells (U87-MG and T98-G cells). The quadrants Q1 stands for necrosis, Q2 for late apoptosis Q3 for viable cells, and Q4 for late apoptosis. Apoptosis (Q2+Q4) % of naringin, TMZ, and naringin + TMZ combination were 55.3%, 55.8%, and 70.8% compared with 40.6% of untreated control, respectively in U87-MG cells. Apoptosis (Q2+Q4) % of naringin, TMZ, and naringin + TMZ combination were 47.2%, 41.3%, and 48.6% compared with 33.3% of untreated control, respectively in T98-G cells. Not much difference was found in the percentage of apoptosis between the control and TMZ groups in TMZ resistant cells, whereas a relative percentage increase was seen in the naringin TMZ, and naringin + TMZ combination groups as compared to the control group. Apoptosis results show that naringin synergistically stimulated the apoptotic effects of TMZ in both TMZ sensitive and resistant cells. Moreover, we discovered apoptosis as a possible naringin target by network pharmacology.

### 3.9 Naringin and TMZ-induced G0/G1 cell cycle arrest in U87-MG and T98-G cells

To further investigate the effect of naringin and TMZ on cell cycle in U87-MG and T98-G cells, cell cycle analysis was performed using PI. According to Figure 7I-L, the percentage of U87-MG cells in the G0/G1 phase increased from 59.3% in control to 64.3%, 65.1%, and 67.5% with pretreatment of naringin (121.3 μM), TMZ (212.5 μM), and naringin + TMZ (38.14 + 38.14 μM), respectively. According to Figure 7M-P, the percentage of T98-G cells in the G0/G1 phase increased from 54.9% in control to 59.5%, 54.8%, and 67.9% with pretreatment of naringin (243 μM), TMZ (212.5 μM), and naringin + TMZ (243 + 212.5 μM), respectively. Not much difference was found in the percentage of apoptosis between the control and TMZ groups in T98-G cells, whereas a relative percentage increase was seen in the naringin, TMZ and naringin + TMZ combination groups as compared to the control group. Cell cycle results show that when naringin and TMZ were given in combination, there was a significant increase in cell cycle arrest at the G0/G1 phase, showing the chemosensitization of TMZ in TMZ resistance cells.

### 3.10 Naringin, TMZ, and naringin + TMZ combination inhibit DNA repair pathway of glioblastoma

Expression of PARP-1 and MGMT (proteins involved in DNA repair pathway) were detected in T98-G cells by flow cytometry analysis. As demonstrated in Figure 5E, there is not much difference in the expression of PARP-1, and MGMT in the TMZ group compared to the control group with TMZ (212.5 μM) pretreatment showing resistance in T98-G cells. However, PARP-1 and MGMT expression comprised a relatively low percentage in naringin and naringin + TMZ groups as compared to the control group with pretreatment of naringin (243 μM), and naringin + TMZ (243 + 212.5 μM), showing the chemosensitization of resistant cells. Overall, these findings indicate that treatments with naringin and naringin + TMZ combination result in the downregulation of the expression of PARP-1 and MGMT in T98-G cells.

### 3.11 Evaluation of DNA repair and apoptosis pathway by naringin and TMZ through Western blotting

**FIGURE 8:**
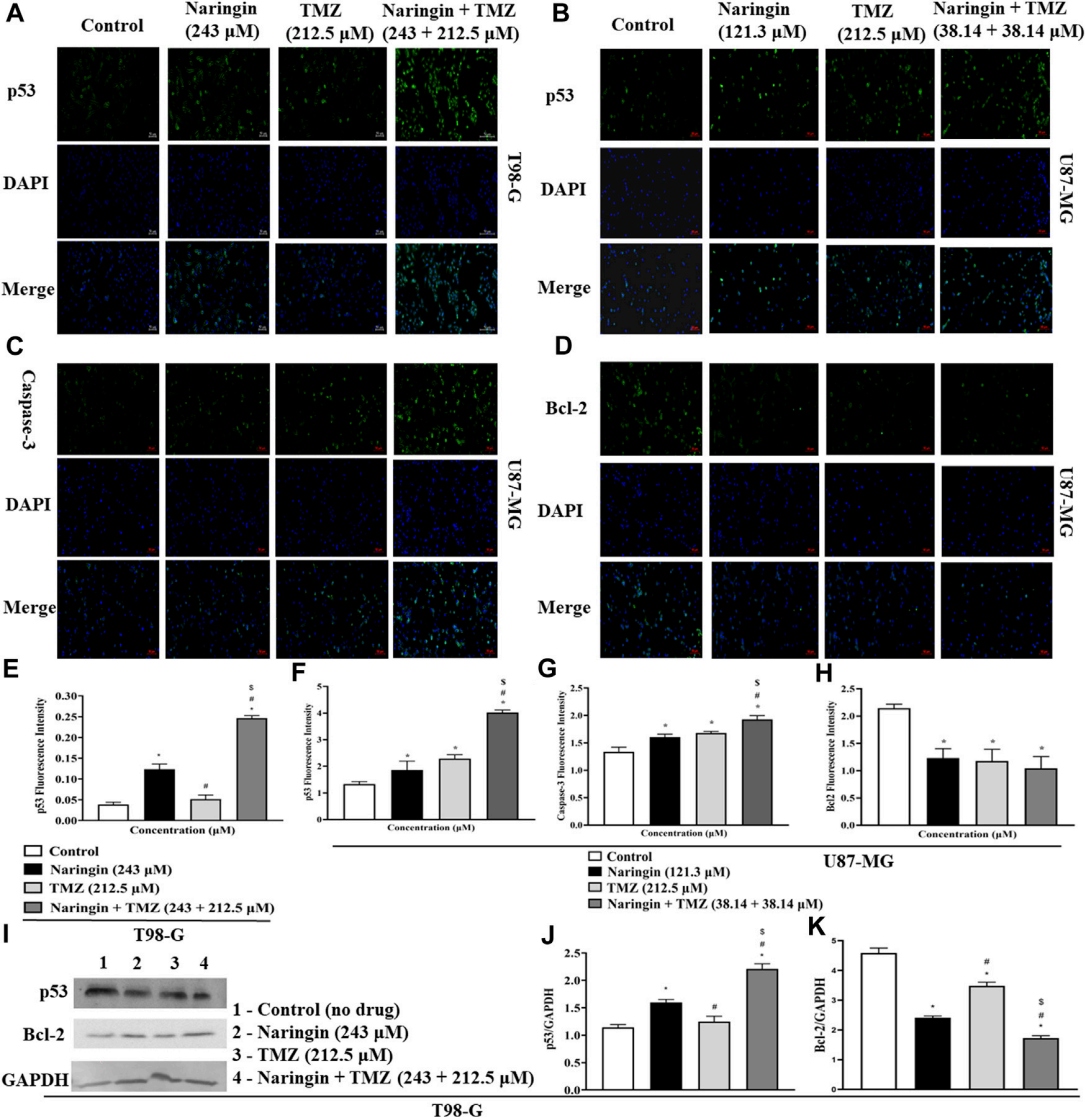
Expression of apoptotic and anti-apoptotic proteins in U87-MG and T98-G cells by Western blotting and ICC. **(A,B)** p53, **(C)** Caspase - 3 **(D)** Bcl-2, **(E–H)** Bar graph showing the fluorescence intensity of p53, caspase-3 and BCL-2. **(I)** Lanes 1, 2, 3, and 4 are the control (no drug), naringin (243 μM), TMZ (212.5 μM), and naringin + TMZ (243 + 212.5 μM) groups, respectively, in the presentative Western blot picture of several proteins. The loading control used was GAPDH. **(J,K)** Bar graph showing the expression of p53 and Bcl-2. Scale bars: 50 µm. For quantification of blots and images ImageJ software was used. Results were presented as mean ± SD, n = 3. The D'Agostino-Pearson omnibus *post hoc* test was used to analyze the results’ normality of distribution. One-way ANOVA and Tukey’s multiple comparison tests were used for every possible comparison between the study groups. (*) *p* < 0.05, vs control; (#) *p* < 0.05 vs naringin; ($) *p* < 0.05 vs TMZ. Abbreviation: ICC - Immunocytochemistry, PARP-1 - Poly [ADP-ribose] polymerase 1, Bcl2 - B-cell lymphoma 2, TMZ - Temozolomide.

**FIGURE 9:**
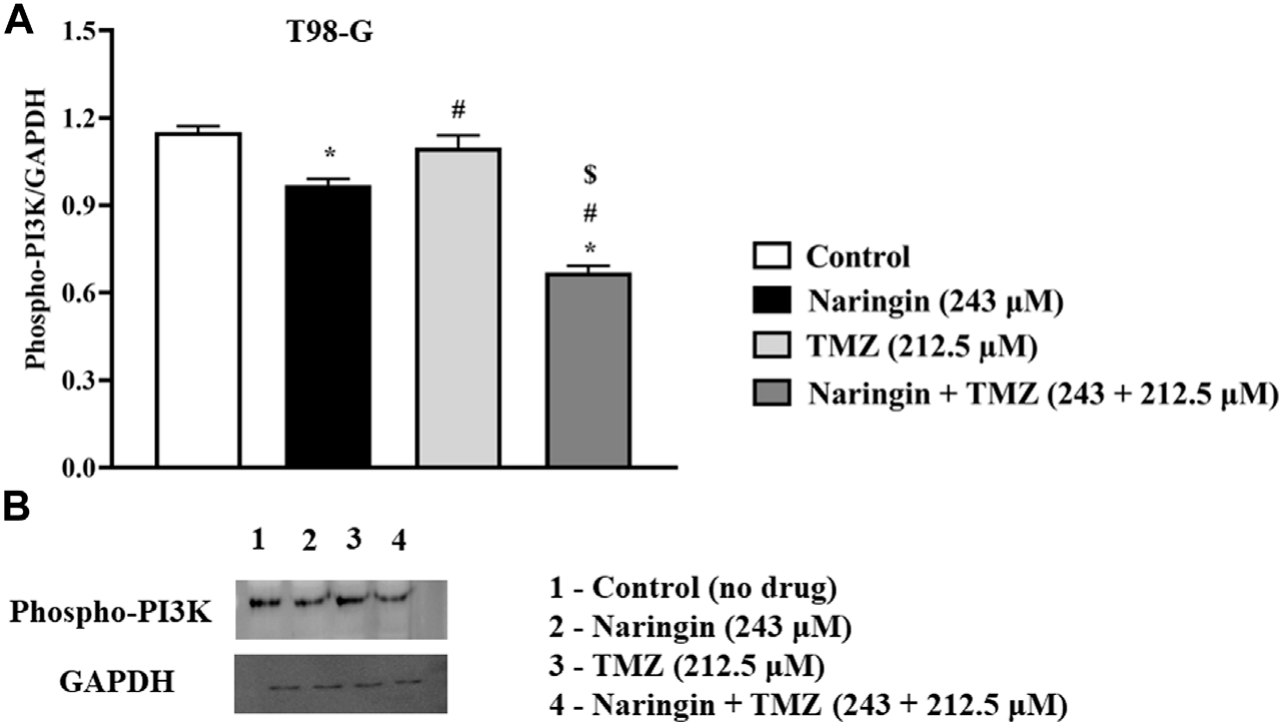
Expression of Phospho PI3K protein in T98-G cells by Western blotting. **(A)** Bar graph showing the expression of Phospho PI3K. **(B)** Lanes 1, 2, 3, and 4 are the control (no drug), naringin (243 μM), TMZ (212.5 μM), and naringin + TMZ (243 + 212.5 μM) groups, respectively, in the presentative Western blot picture of Phospho PI3K protein. The loading control used was GAPDH. For quantification of blots ImageJ software was used. Results were presented as mean ± SD, n = 3. The D'Agostino-Pearson omnibus *post hoc* test was used to analyze the results’ normality of distribution. One-way ANOVA and Tukey’s multiple comparison tests were used for every possible comparison between the study groups. (*) *p* < 0.05, vs control; (#) *p* < 0.05 vs naringin; ($) *p* < 0.05 vs TMZ. Abbreviation: Phospho PI3K - Phospho Phosphatidylinositol 3-kinase, TMZ - Temozolomide.

To explore the role of naringin and TMZ in DNA repair and apoptosis pathways, Western blotting was utilized to analyze the expression study of PARP-1 (Figure 5D), Bcl2, p53 (Figure 8I), and Phospho-PI3K (Figure 9) in T98G cells. The expression of PARP-1 Bcl2, Phospho-PI3K in T98G cells is significantly (*p* < 0.05) reduced in the treatment group with pretreatment of TMZ at 212.5 μM, naringin at 243 μM, and naringin + TMZ at 243 + 212.5 μM as compared to the control group. The expression of p53 is significantly (*p* < 0.05) increased in the treatment group with pretreatment of naringin at 243 μM, and naringin + TMZ at 243 + 212.5 μM as compared to the control group. However, there is no change in the expression of p53 in the TMZ group compared to the control group with pretreatment of TMZ at 212.5 μM. The loading control used was GAPDH. For the quantification of blots, ImageJ software was used. (Un-cropped images of the original blot are available in the Supplementary Material as Supplementary Figure S1).

### 3.12 Immunocytochemistry

To prove that treatment of U87-MG and T98-G cells causes cell death by apoptosis and increases the levels of apoptotic markers p53 and caspase-3 while decreases the level of antiapoptotic markers (bcl2), ICC was carried out. Figure 8A demonstrates in T98-G cells, more cells express significantly (*p* < 0.05), in p53, after treatment with naringin at 243 μM and naringin + TMZ at 243 + 212.5 μM. However, there is no change in the expression of p53 in the TMZ group compared to the control group with TMZ (212.5 μM) pretreatment. Figure 8(B-D) demonstrates in U87-MG cells, more cells express significantly (*p* < 0.05), in p53 and caspase-3 while less cells express significantly (*p* < 0.05), in bcl2 after treatment with naringin at 121.3 μM, TMZ at 212.5 μM, and naringin + TMZ at 38.14 + 38.14 μM (U87-MG cells). These data indicate that treatments with naringin + TMZ combination result in upregulation of the expression of apoptotic protein (p53 and caspase-3) and downregulation of antiapoptotic protein (bcl2).

### 3.13 Analyzing the biochemical effects of naringin and TMZ treatment in T98-G cells using LC-HRMS-based metabolomics study

**FIGURE 10:**
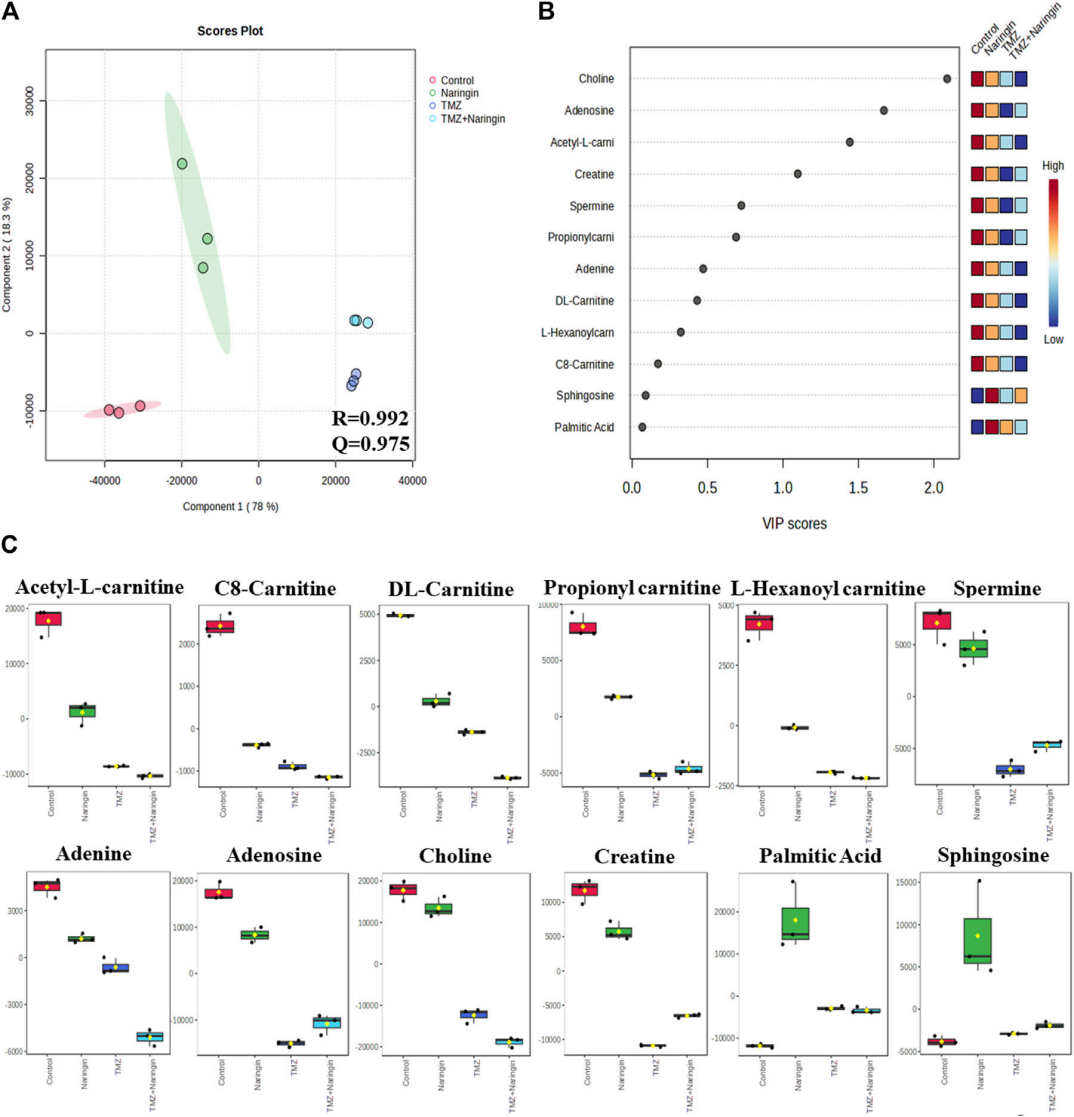
The 2D PLS-DA plot, VIP score analysis, and a box-cum-whisker plot of altered metabolites. **(A)** A two-dimensional OPLS-DA was used in this study to assess the spectrum score plot. The final score plot was produced by a thorough analysis that included every group that was the subject of the study. The red circle represents the control group, the green circle represents naringin (243 μM), the violet circle represents TMZ (450.2 μM), and the blue circle represents naringin + TMZ (243 + 212.5 μM). **(B)**. The process of modeling the whole PLS-DA plot data matrix yields potentially differentiating metabolites that are then arranged in ascending order of VIP score to highlight their discerning potential. **(C)** A box-cum-whisker plot examines the metabolic changes brought about by naringin (243 μM), TMZ (450.2 μM), and naringin + TMZ (243 + 212.5 μM) treatment. The changes seen in the quantitative patterns of metabolites can be visualized using box-cum-whisker plots. The interquartile ranges are displayed as box plots, where the boxes stand in for these ranges. A horizontal line within the box represents the median, and the bottom and higher box bounds correspond to the 25th and 75th percentile values, accordingly. The 5th and 95th percentile values are shown by the lower and upper whiskers, accordingly. A one-way ANOVA test (*p* < 0.05) is used to analyze the continuous data sets, which are all provided as the mean ± SD; n = 3; R2: goodness of fit; Q2: goodness of prediction; VIP: 1,000 Variable importance on projections; OPLS-DA: orthogonal-partial-least-squares-discriminant-analysis; PLS-DA: partial-least-squares-discriminant-analysis.

**FIGURE 11:**
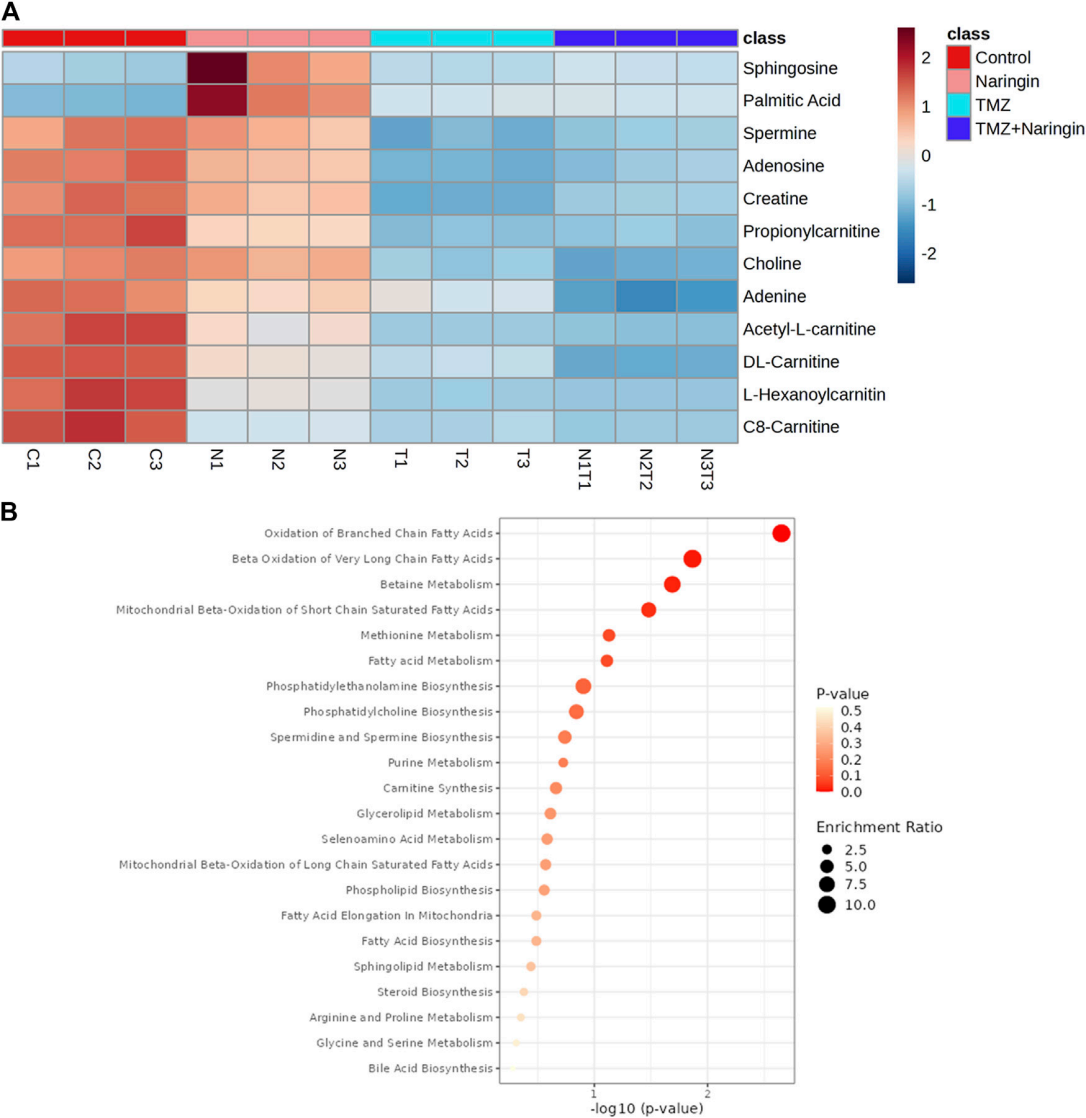
Heatmap illustration and associated metabolic pathways. **(A)** A heat map that shows how the top 12 different metabolites—selected using the *t*-test/ANOVA—are grouped hierarchically. Red (upregulation) and blue (downregulation) colors indicate the degree of change. **(B)** Using the pathway enrichment analysis with the KEGG database, commonly chosen metabolites (12) were categorized into various metabolite pathways. Red denotes low q-values and orange denotes high q-values; the size of the circle reflects the proportion of enriched metabolites. All data sets are shown as mean ± SD; n = 3.

Samples of cells from the treatment and control groups were subjected to LC-HRMS analysis. Using multivariate statistical experimentation, the effects of naringin (243 μM), TMZ (450.2 μM; IC50 of TMZ in T98-G cells), and naringin + TMZ (243 + 212.5 μM) treatments on these metabolic changes were compared to the metabolic changes in the control group. As seen, normalized data from the binned spectral spectrum were examined to create the PLSDA plot (Figure 10A). The PLS-DA score plot makes it evident that the control group has significant metabolic changes by explicitly distinguishing it from the naringin, TMZ, and naringin + TMZ treatment groups. There is a noticeable tendency for the naringin + TMZ group to cluster and normalize when compared to naringin, TMZ, and naringin + TMZ. With R2 = 0.999 and Q2 = 0.998, accordingly, the VIP score plots in Figure 10B show how various groups may be distinguished from one another. Together, the 2D PLS-DA plot and VIP score analyses showed significant differences in the metabolic profiles of the various groups. The initial top 12 metabolite sensitizers were carefully chosen following the creation of a statistically significant threshold of VIP values by the PLS-DA model whose value surpasses 1.0. At a significance level of *p* < 0.05, it was determined that the *p*-values acquired from the one-way ANOVA for the regulated peak matrix were statistically significant. To determine the degree of variance displayed by these highly variable metabolites among the several groups under examination, the log-2 fold change technique was utilized. If the *p*-value is less than 0.05, then the findings are statistically significant. The metabolic plots created using the box cum-whisker technique (Figure 10C) show the variation in quantitative terms displayed by a number of the identified discriminating metabolites. Chromatogram (Supplementary Figure S2), fold change and *p*-value (Supplementary Table S1) for metabolites are shown in Supplementary Material). Figure 10C shows that of the three treatment groups, the naringin + TMZ treatment was the most significant, with all of the (naringin, TMZ, and naringin + TMZ) treated groups exhibiting amelioration of metabolic abnormalities towards normalization. The metabolomic profiles of the naringin, TMZ, and naringin + TMZ treated groups were comparable to those of the control group, according to the heat mapping of all data sets (Figure 11A). After receiving naringin, TMZ, and naringin + TMZ treatment, a number of particular metabolites related to glioblastoma had significant changes in the control group but varied degrees of reversals afterward. These individual metabolites may be involved in a number of important pathways (Figure 11B). Among the main metabolic pathways impacted by naringin, TMZ, and naringin + TMZ administration within our dataset were classified into three metabolic pathway modules, i.e., Oxidation pathway (fatty acid), metabolism pathways (betaine, methionine, fatty acid, purine, glycerolipid, selenoamino acid sphingolipid, arginine, proline, glycine, and serine), and biosynthesis pathways (phosphatidylethanolamine, phosphatidylcholine, spermidine, spermine, and carnitine) were among the major metabolic pathways affected by naringin, TMZ, and naringin + TMZ administration within our dataset.

## 4 Discussion

Glioblastoma is the most fatal type of primary invasive brain tumor. At present, the three principal clinical treatment strategies are surgical removal of the tumor, radiotherapy, and treatment with TMZ. However, TMZ resistance frequently limits the ability to treat patients adequately (Bisht et al., 2022). Bioflavonoids, a common class of polyphenolic compounds, are currently being investigated as experimental medicinal agents in a variety of fields (Mahmud et al., 2023). Naringin, a flavanone-7-O-glycoside, has been a popular candidate in the therapeutics arena due to its powerful anti-cancer, antioxidant, metal-chelating, and lipid-lowering effects (Raja Kumar et al., 2019; Salehi et al., 2019). To explore the targets and pathways of naringin in glioblastoma network pharmacology was used. By comparing the targets of naringin and glioblastoma, we discovered that glioblastoma shares 51 targets with naringin. The PPI network was constructed to show the functional interactions between the target proteins. The findings of the PPI network analysis suggested that the key targets of naringin in the chemosensitization of glioblastoma would be PARP-1, MGMT, and caspases. The functional enrichment analysis revealed that these targets were significantly enriched in important pathways such as p53 signaling, apoptosis, and DNA sensing. In this study, first, an MTT test was performed to assess the cytotoxicity and IC50 values of naringin and TMZ. The respective IC50 values for naringin, TMZ, and naringin + TMZ on U87-MG cells were 121.3, 212.5, and 38.14 + 38.14 μM respectively in U87-MG cells. The IC50 values for naringin and TMZ were 243 and 450.2 μM on T98-G cells. Later, the IC50 value of TMZ on U87-MG was taken for further study to show the TMZ resistance in T98-G cells. There is no significant difference found between the control and TMZ groups showing the TMZ resistance in T98-G cells at the concentration of 212.5 μM. However, in naringin + TMZ combination group (243 + 212.5 μM) showed the 76% killing of glioblastoma cells, showed the sensitization of TMZ against TMZ resistance cells. The dynamic and multifaceted mechanism of wound healing involves several cellular processes intended to repair and regenerate the injured skin area. The development of a clot is the initial stage of this procedure, which is followed by other occurrences including blood circulation, the arrival of inflammatory immune cells, as well as the movement of cells, their proliferation, and differentiation to heal the wound (Nosrati et al., 2021). Naringin and TMZ’s ability to inhibit the regenerative process in resistant cells by wound healing assay was evaluated. A significant difference between the control and the naringin and naringin + TMZ combination group was found, which showed that naringin and TMZ inhibit the migration activity in resistant cells.

The apoptotic effect of the naringin and TMZ was assessed by annexin V, Western blotting, and ICC in both cell lines (U87-MG and T98-G). The apoptotic effect by annexin V showed the highest % of apoptosis in the naringin, TMZ, and naringin + TMZ groups in U87-MG cells and TMZ and naringin + TMZ groups in T98-G cells. Further, Western blotting and ICC were performed to evaluate the apoptosis pathway. In the result, it was found that the expression of p53 and caspase-3 is significantly increased in naringin, TMZ, and the naringin + TMZ group in U87-MG cells and the expression of p53 is significantly increased in TMZ and naringin + TMZ group in T98-G cells shows the apoptosis in the treatment group. Similarly, a significant decrease in the expression of bcl2 was found compared to the control group in the naringin, TMZ, and naringin + TMZ group in U87-MG cells and in naringin and naringin + TMZ group in T98-G cells. These results show that treatments with naringin, TMZ and naringin + TMZ combination result in upregulation of the expression of the p53 and caspase-3 and downregulation of bcl2 in U87-MG cells and treatments with naringin and naringin + TMZ combination result in upregulation of the expression of the p53 and downregulation of bcl2 in T98-G cells. Further cell cycle arrest analysis was performed by FACS using PI. There was no difference found between the control and TMZ groups in T98-G cells cell cycle arrest. However, in the naringin and naringin + TMZ group, a significant increase in cell cycle arrest was found at the G0/G1 phase, showing the chemosensitization of TMZ in TMZ resistance cells. In U87-MG cells, naringin, TMZ and naringin + TMZ group, a significant increase in cell cycle arrest was found at the G0/G1 phase.

PARP is a type of enzyme that is involved in the BER pathway as well as the MGMT pathway by directly interacting with and eventually PARylating MGMT after being treated with TMZ to remove O6-methylguanine (O6-MetG) adducts from the damaged region of DNA. Second, PARP functions as a sensor, activating the BER response pathways. PARP inhibitors reduce PARP-MGMT binding or PARylation of MGMT, lowering MGMT function and blocking O6-MetG repair. As a result, the MGMT function is diminished, leading to TMZ sensitivity and providing evidence for sensitization (Bisht et al., 2022). Here, in this study, the expression of PARP-1 and MGMT was evaluated and it was found that PARP-1 and MGMT expression comprised a relatively low percentage in naringin and naringin + TMZ groups showing that naringin inhibited the expression of PARP-1 and MGMT and in combination the expression is relatively less in comparison to naringin leading to chemosensitization of resistance cells (T98-G). MGMT expression, however, was not detected in U87-MG cells. Inhibited PARP-1 expression was seen in the naringin, and naringin + TMZ groups.

One well-known signaling mechanism that controls many distinct cellular functions, such as migration, proliferation, differentiation, and apoptosis, is the PI3K/AKT pathway (Porta et al., 2014). Anti-cancer medications have been shown in earlier research to induce apoptosis by inhibiting the PI3K/AKT signaling pathway (Yang et al., 2018; Zhu et al., 2020). Naringin stops cell division and triggers apoptosis in colorectal cancer by inhibiting the PI3K/AKT signaling pathway (Cheng et al., 2020). Naringin causes apoptosis and suppresses thyroid cancer cell growth by targeting this pathway (Zhou et al., 2019). The current study’s findings demonstrated that TMZ with naringin might significantly increase T98-G cell apoptosis by blocking the PI3K/AKT signaling pathway.

Furthermore, a quantitative understanding of the glioblastoma’s metabolic profile before and after naringin, TMZ, and naringin + TMZ treatments is provided by the LC-HRMS-based metabolomics analysis. Acknowledging the altered metabolites in the glioblastoma prognosis could help with the treatment of tumors and early diagnosis. Through statistical multivariate data interpretation via MetaboAnalyst 6.0 (Bang et al., 2022), a metabolic profile study was carried out to look at the metabolic changes caused by tumorous cells and how naringin, TMZ, and naringin + TMZ perturbation metabolic profiles. Cancerous cells can spread more easily and aggressively due to metabolic plasticity. A vital facilitator of cancer-associated metabolic plasticity, the carnitine system links important metabolic pathways, variables, and metabolites to provide cancer cells with the energy and biosynthetic resources they need (Melone et al., 2018). The C8-carnitine, L-Hexanoylcarnitine, DL-carnitine, Acetyl-L-carnitine, and Propionylcarnitine levels in our study were higher in the control group than in the treated groups. The current results are consistent with other research that has been published and has shown that the carnitine system mediates cancer metabolic plasticity (Melone et al., 2018).

According to recent studies, cancer causes major alterations in the metabolism of choline, which increases the body’s need for choline and its metabolites. Because cancer cells proliferate quickly, more phosphatidylcholine (PtdCho) must be produced for new cell membranes to develop. Moreover, choline uptake and consumption are enhanced in cancer cells due to modified signaling pathways. The development of tumors, treatment resistance, and the initial stages of cancer have all been connected to these alterations in choline metabolism (Yao et al., 2023). In our study, it was discovered that choline levels reduced after treatments with naringin, TMZ, and naringin + TMZ.

Elevated creatine levels are associated with faster cancer progression in the majority of cancer types. Even while creatine has been shown to have anticancer properties, recent research has shown that creatine accelerates the growth of cancer, especially when it comes to metastasis and invasion (Kazak and Cohen, 2020). The control group in our study had greater creatine levels than the groups that received treatment.

Since purines are fundamental building blocks of nucleotides in the growth of cells, poor purine metabolism is linked to the development of cancer. It has been shown that tumor cells contain significant concentrations of purine metabolites (Yin et al., 2018). In the present study, the treated groups’ levels of adenine and adenosine were lower than those of the control group.

Human cells contain millimolar amounts of the polyamines putrescine, spermidine, and spermine, which are polycationic alkylamines. Significantly, these highly charged, low molecular mass molecules play a key role in numerous crucial steps of cell development and sustenance. In general, the dysregulation of polyamine metabolism in cancer suggests that higher polyamine levels are required for growth and tumor development (Casero et al., 2018). In the present study, spermine levels were shown to have decreased following treatment in comparison to the control group.

Ceramide, sphingosine, and sphingosine 1-phosphate (S1P) are important modulators of cellular growth and apoptosis. These three sphingolipids are interchangeable: ceramide, and sphingosine 1-phosphate (S1P). Sphingosine and ceramide have been linked to apoptosis and growth arrest when exposed to various stimuli of stress, such as chemotherapy and radiation. Unlike sphingosine and ceramide, S1P increases the survival of cells in the presence of apoptotic stressors (Cuvillier, 2008). The control group in the present study had higher sphingosine levels than the groups that received treatment.

Recent research in pharmacology has shown that palmitic acid (PA) has immune-stimulating, antioxidant, and anti-inflammatory properties. Its anti-tumor activities include causing tumor cells to undergo apoptosis, preventing tumor cell growth, preventing metastasis and invasion, increasing chemotherapy sensitivity, and boosting immune system performance (Wang et al., 2023). In treated groups, there has been an upregulation of PA, which might indicate the beginning of apoptosis.

## 5 Conclusion

**FIGURE 12:**
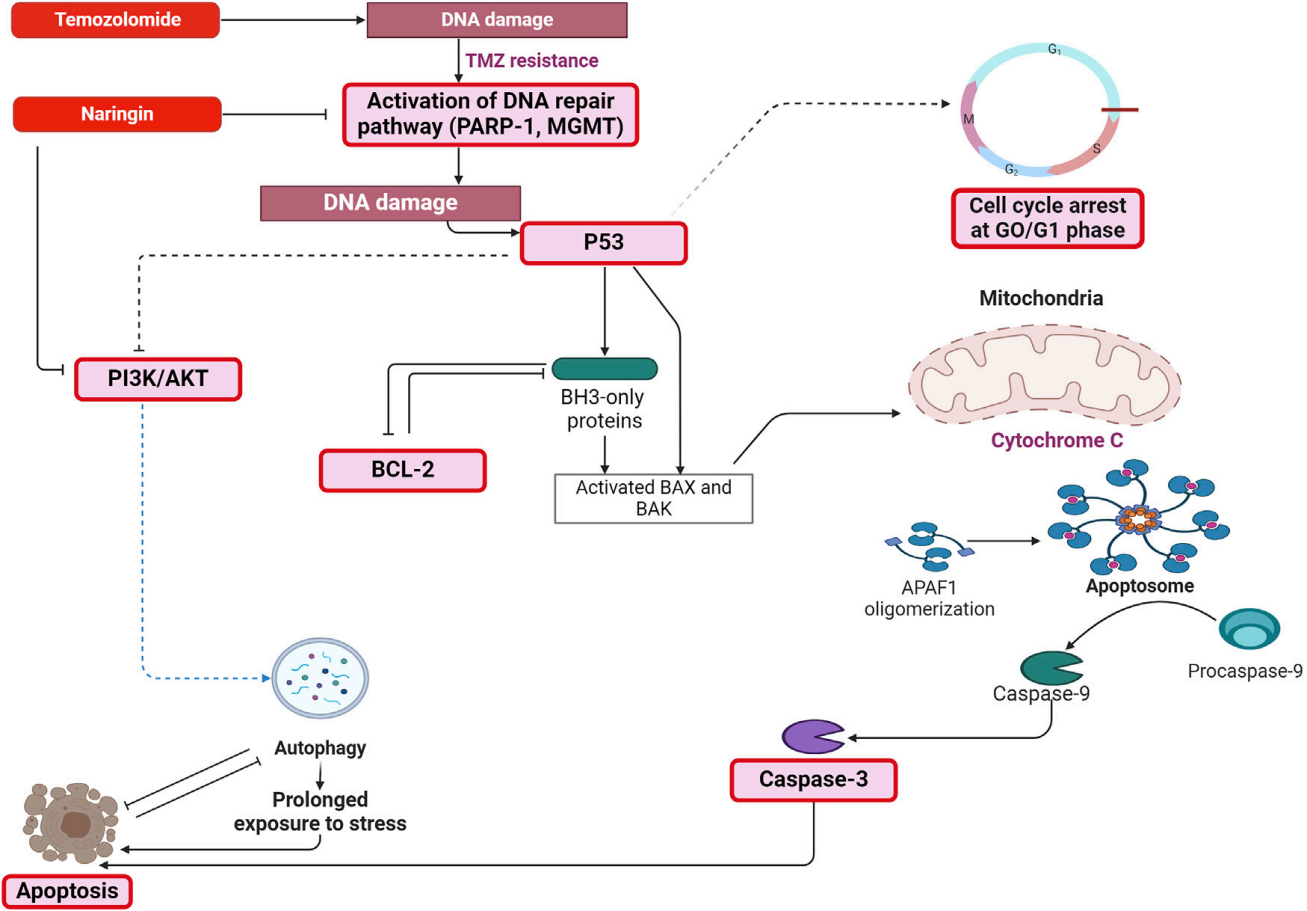
Mechanism depicting the pathway through which naringin in combination with TMZ causes chemosensitization of TMZ towards glioblastoma cells by inhibiting the DNA repair pathway (PARP-1 and MGMT) and causing apoptosis in tumor cells by increasing the expression of p53, caspase-3, PI3K and by decreasing the expression of Bcl-2. Abbreviation: TMZ - Temozolomide, PARP-1 - Poly [ADP-ribose] polymerase 1, MGMT - O-6-Methylguanine-DNA Methyltransferase, Bcl2 - B-cell lymphoma 2, Phospho PI3K - Phospho Phosphatidylinositol 3-kinase, Bax - Bcl-2 Associated X-protein, Bak - B-cell lymphoma 2 (BCL-2) antagonist/killer, APAF-1 - Apoptotic protease activating factor 1.

The naringin + TMZ treatment group showed greater prominence during the metabolic alteration among all treatment groups. The precise mechanism of death in T98-G cells after exposure to naringin, TMZ, and naringin + TMZ could therefore be clarified by conducting focused investigations on prominent metabolites (such as fatty acids, polyamines, and nucleotides) being involved in different metabolic pathways (such as beta-oxidation of fatty acids, fatty acid metabolism, Purine metabolism, and sphingolipid metabolism, etc.). In conclusion, the above results demonstrated that naringin in combination with TMZ causes chemosensitization of TMZ towards glioblastoma cells by inhibiting the DNA repair pathway (PARP-1 and MGMT) and causing apoptosis in tumor cells (Figure 12). Thus, the insights of this study will help to overcome the drawback of TMZ resistance in glioblastoma by producing the synergistic effect of TMZ by combining it with naringin. Nevertheless, more research on animals must be performed to verify its therapeutic potential. The results should pave the way for the building of plant-based nutritional resources with anti-cancer properties in the field of nutritional research.

## Data Availability

The original contributions presented in the study are included in the article/Supplementary Material, further inquiries can be directed to the corresponding author.
