# Supplementary material for: Naringin and temozolomide combination suppressed the growth of glioblastoma cells by promoting cell apoptosis: network pharmacology, in-vitro assays and metabolomics based study
Source: Front Pharmacol. 2024 Jul 30;15:1431085. doi: 10.3389/fphar.2024.1431085 (PMC11325085; doi:10.3389/fphar.2024.1431085)
Supplement: Supplementary file 1 [file Presentation1.zip › Supplementary files/SUPPLEMENTARY MATERIALS.pdf]

SUPPLEMENTARY MATERIALS

GAPDH

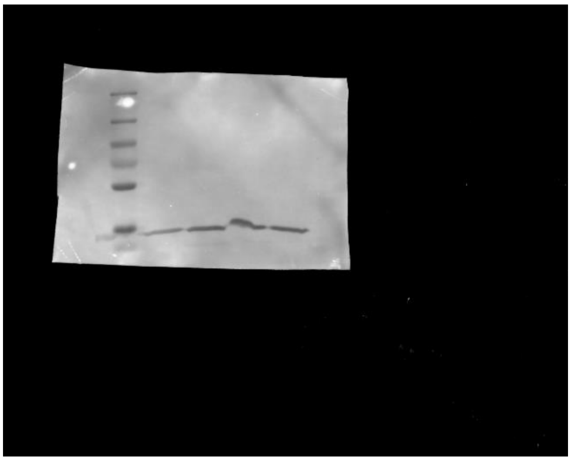

PARP-1

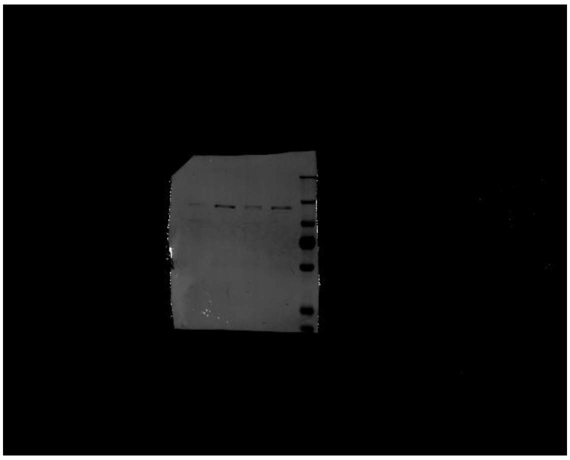

P53

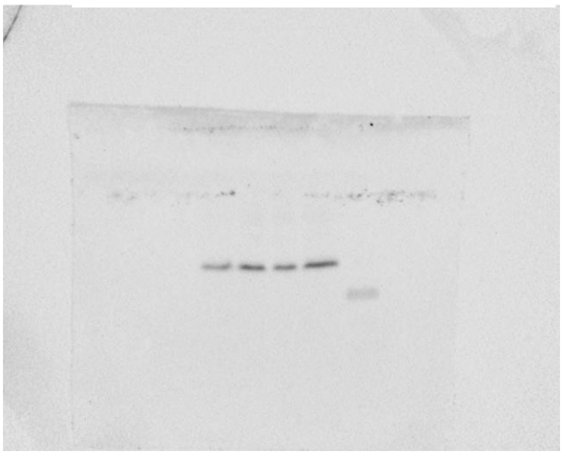

BCL2

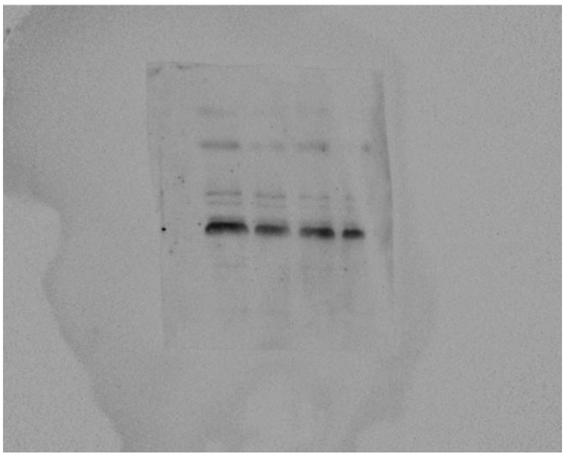

GAPDH

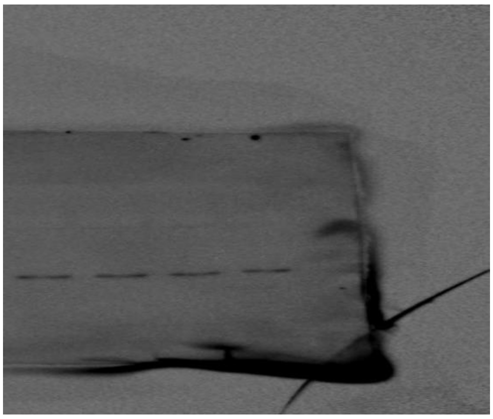

Phospho-PI3K

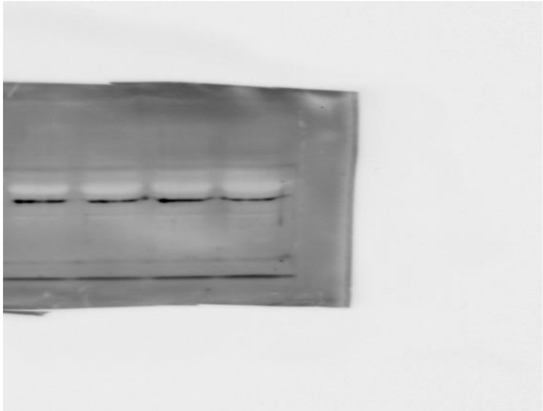

Supplementary Figure 1: Un-cropped images of original blots

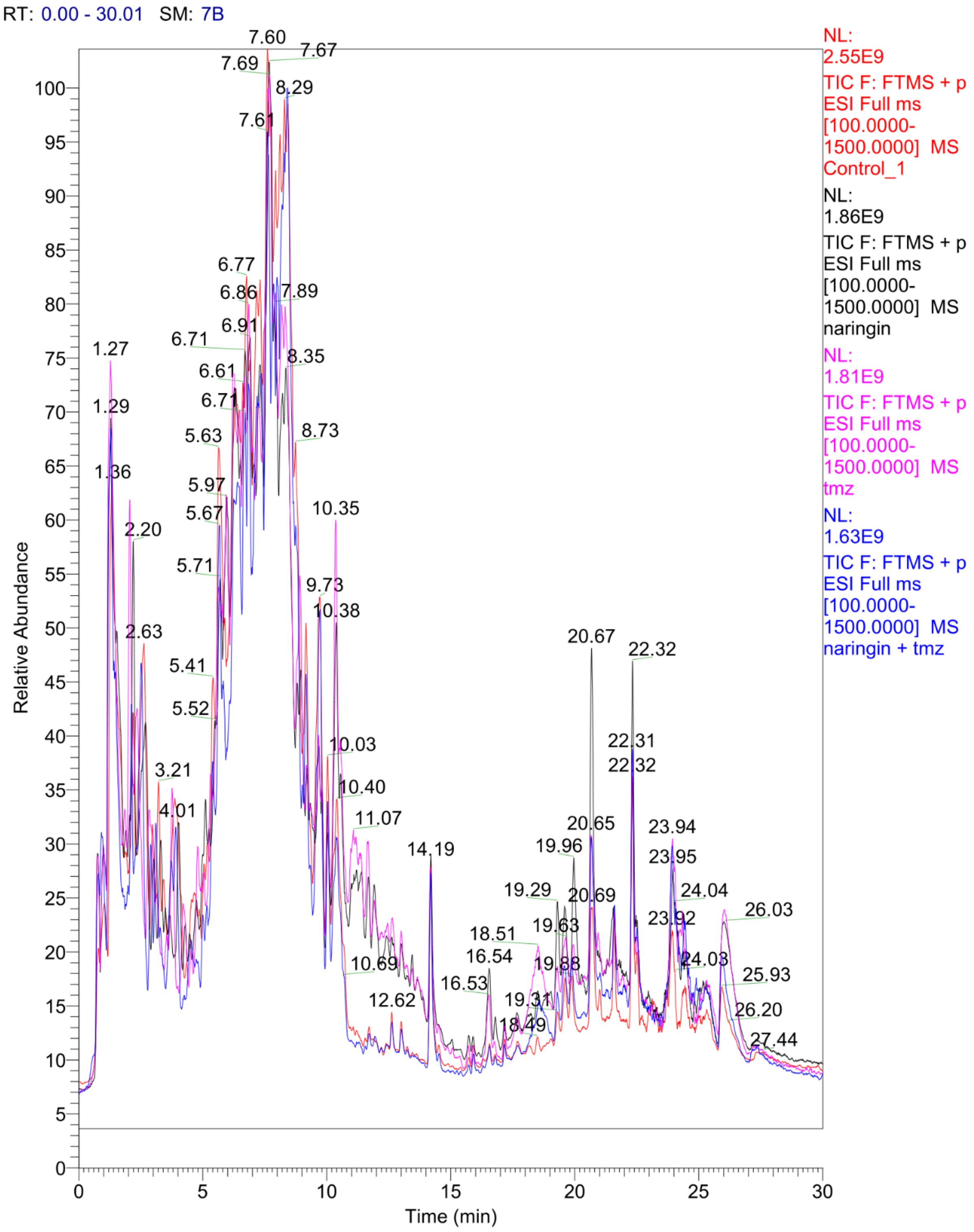

Supplementary Figure 2: Representative Extracted ion chromatogram for control, naringin, TMZ, and naringin + TMZ are shown in figure.
